# Genetic diversity of *Escherichia coli* in gut microbiota of patients with Crohn’s disease discovered using metagenomic and genomic analyses

**DOI:** 10.1186/s12864-018-5306-5

**Published:** 2018-12-27

**Authors:** Alexander V. Tyakht, Alexander I. Manolov, Alexandra V. Kanygina, Dmitry S. Ischenko, Boris A. Kovarsky, Anna S. Popenko, Alexander V. Pavlenko, Anna V. Elizarova, Daria V. Rakitina, Julia P. Baikova, Valentina G. Ladygina, Elena S. Kostryukova, Irina Y. Karpova, Tatyana A. Semashko, Andrei K. Larin, Tatyana V. Grigoryeva, Mariya N. Sinyagina, Sergei Y. Malanin, Petr L. Shcherbakov, Anastasiya Y. Kharitonova, Igor L. Khalif, Marina V. Shapina, Igor V. Maev, Dmitriy N. Andreev, Elena A. Belousova, Yulia M. Buzunova, Dmitry G. Alexeev, Vadim M. Govorun

**Affiliations:** 1 Federal Research and Clinical Centre of Physical-Chemical Medicine, Malaya Pirogovskaya 1a, Moscow, 119435 Russia; 2 Moscow Institute of Physics and Technology, 9 Institutskiy per., Dolgoprudny, Moscow Region, Russian Federation 141700; 3 ITMO University, 49 Kronverkskiy pr, Saint-Petersburg, Russian Federation 197101; 4 Kazan Federal University, 18 Kremlyovskaya St., Kazan, Russian Federation 420008; 5 Moscow Clinical Scientific Center, 86 Shosse Entuziastov St., Moscow, Russian Federation 111123; 6 Clinical and Research Institute of Emergency Children’s Surgery and Trauma, 22 Bolshaya Polyanka St., Moscow, Russian Federation 119180; 7 State Scientific Center of Coloproctology, 2 Salam Adil St., Moscow, Russian Federation 123423; 8 Moscow State University of Medicine and Dentistry, Build. 6, 20 Delegatskaya St., Moscow, Russian Federation 127473; 9 Moscow Regional Research and Clinical Institute, 61/2 Shchepkina str, Moscow, Russian Federation 129110; 10 M.M. Shemyakin - Yu.A. Ovchinnikov Institute of Bioorganic Chemistry of the Russian Academy of Sciences, 16/10 Miklukho-Maklaya St., Moscow, Russian Federation 117997

**Keywords:** Gut microbiota, Crohn’s disease, *Escherichia coli*, Gene content, Inflammatory bowel diseases, Metagenomics, Pangenome

## Abstract

**Background:**

Crohn’s disease is associated with gut dysbiosis. Independent studies have shown an increase in the abundance of certain bacterial species, particularly *Escherichia coli* with the adherent-invasive pathotype*,* in the gut. The role of these species in this disease needs to be elucidated.

**Methods:**

We performed a metagenomic study investigating the gut microbiota of patients with Crohn’s disease. A metagenomic reconstruction of the consensus genome content of the species was used to assess the genetic variability.

**Results:**

The abnormal shifts in the microbial community structures in Crohn’s disease were heterogeneous among the patients. The metagenomic data suggested the existence of multiple *E. coli* strains within individual patients. We discovered that the genetic diversity of the species was high and that only a few samples manifested similarity to the adherent-invasive varieties. The other species demonstrated genetic diversity comparable to that observed in the healthy subjects. Our results were supported by a comparison of the sequenced genomes of isolates from the same microbiota samples and a meta-analysis of published gut metagenomes.

**Conclusions:**

The genomic diversity of Crohn’s disease-associated *E. coli* within and among the patients paves the way towards an understanding of the microbial mechanisms underlying the onset and progression of the Crohn’s disease and the development of new strategies for the prevention and treatment of this disease.

**Electronic supplementary material:**

The online version of this article (10.1186/s12864-018-5306-5) contains supplementary material, which is available to authorized users.

## Background

Gut microbiota disequilibrium is among the accepted hallmarks of Crohn’s disease (CD). Although the aetiology of this disease is still not completely clear, in addition to genetics and lifestyle, the microbial community structure is among the factors contributing to its pathology [1]. The importance of understanding the role of microbiota in CD and the promising opportunity of the therapeutic modulation of its composition have been recently demonstrated by successful CD treatment via faecal transplantation in pilot studies (for example, in adult [2] and paediatric [3] patients; a recent meta-analysis includes 11 studies [4]).

Using metagenomic high-throughput sequencing, the changes in the structure and function of gut microbiota in CD can be assessed. Surveys of 16S rRNA and “shotgun” formats have revealed a decrease in the total species diversity and abundance of butyrate-producing species accompanied by the growth of Proteobacteria and other opportunist species [5–8]. An analysis of “shotgun” sequencing data has provided evidence of shifts in the functional composition, including the increased presence of genes linked to inflammation, oxidative stress (including glutathione transport) and amino acid degradation [5]. Changes in microbial metabolism in the ileum of CD patients have also been reported based on proteomic studies [9]. Correlation analyses of microbial gene abundance levels have shown that the decrease in community richness in CD is likely due to the extinction of understudied species without representative reference genomes [10]. In addition to the changes in the microbial community, the gut virome composition has been found to be abnormal in patients with CD [11, 12].

The metagenomic profiling of microbiota in patients with CD is complicated by certain methodological challenges. Antimicrobial, anti-inflammatory and other types of treatments represent independent factors that strongly influence the community composition [7, 13]. To distinguish the effect of medical prescriptions from the effect caused by the disease, the metagenome has been examined in treatment-naive patients, e.g., children [14]. However, in the above-mentioned study, the ileal microbiota was profiled using 16S rRNA sequencing of biopsy samples, which limited the assessment of the gene content (biopsy samples are not directly applicable due to the absolute dominance of human DNA over the microbial fraction [15]). Furthermore, species with highly similar 16S rRNA gene sequences might carry substantially different sets of accessory genes, including groups of genes with special clinical significance, such as antibiotic resistance determinants and virulence factors [16].

To date, no single microbial species has been identified as an unambiguously strong factor contributing to the onset of Crohn’s disease. However, *Escherichia coli*, particularly the adherent-invasive pathotype (AIEC), is among the species often detected in increased abundance in CD patients [17, 18]. This species is a beneficiary of inflammation that is able to persist in the gut mucosa in inflammatory tissue. Specific varieties isolated from CD patients with the AIEC pathotype are able to invade epithelial cells, survive in macrophages [19, 20] and form biofilms [21]. The increase in Proteobacteria in the ileum has been shown to be correlated with the Pediatric Crohn’s Disease Activity Index (PCDAI) [22]. Physiologically, the survival of *E. coli* is linked to an increase in the reactive oxygen species levels and a decline in the populations of commensal bacteria which normally provide a protective effect in the host’s intestine. *E. coli* is not associated with the underlying pathology of CD but rather a marker of the inflammatory process [23]. Although *E. coli* is among the most examined model organisms, the enormous genomic variability of this species poses many questions regarding its functioning in vivo. Its identity and role in CD are widely discussed. In CD, are the *E. coli* present clonal or are there distinct genotypes of the organism present? What are the specific features distinguishing *E. coli* in CD patients from *E. coli* in healthy subjects? Finally, is the species a primary cause of Crohn’s disease contributing to the onset of the disease via interplay with the other risk factors or a secondary “first available” species emerging in an environment of general gut imbalance that exacerbates disease progression?

Here, we attempted to decipher the microbial causal factors of CD at multiple levels, i.e., from the gut community level to the single species level (*E. coli*), by integrating cultivation-independent and -dependent methods. We conducted a “shotgun” metagenomic analysis of gut microbiota in CD using stool and ileal content samples collected from patients with CD at two clinical centres.

## Results and discussion

### Microbiota in Crohn’s disease patients exhibits a range of abnormal community structures

**Fig. 1:**
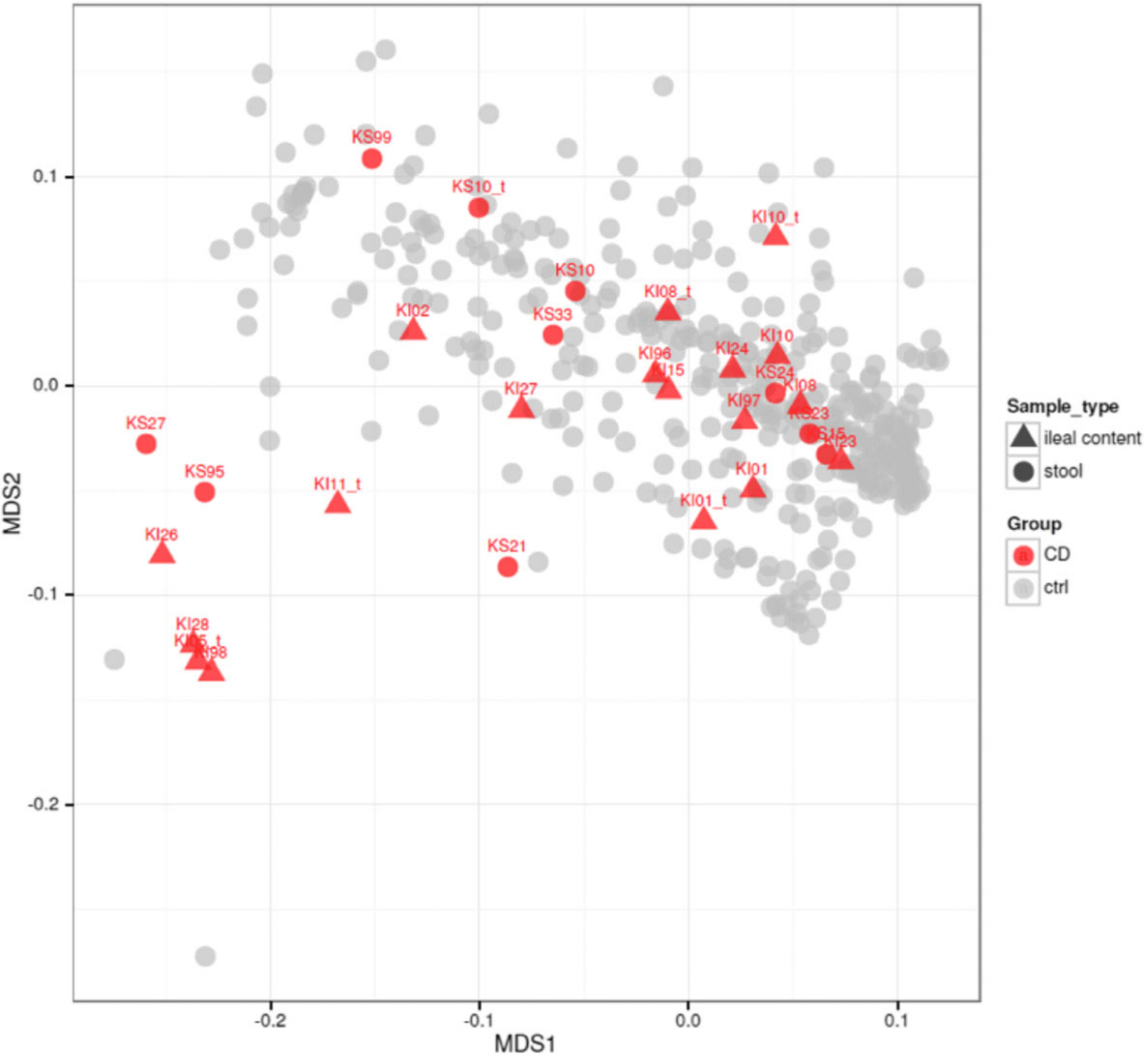
Variation of species-level composition in healthy controls depends on the sample type. Multi-dimensional scaling plot using the whole-genome UniFrac metric. Each point corresponds to a single metagenome; sample type is shown by the shape, while the color shows whether the sample was collected from a CD patient or a subject from an external control group. The external control group included 385 stool metagenomes from healthy Russian, American, Danish and Chinese populations

Generally, stool and ileal metagenomes from the same CD patients tended to be related by taxonomic composition as follows: while the dissimilarity between the paired stool and ileal samples from the same subject was higher than between the replicates of the same sample using different platforms (Bray-Curtis dissimilarity 0.52 ± 0.30 vs. 0.22 ± 0.03, SOLiD vs. Ion Torrent, one-tailed Welch t-test *p* = 0.04), it was significantly lower than the between-pairs variation (0.82 ± 0.15, *p* = 0.05). The species-level profiling of the microbiota showed pronounced dysbiosis as a prevalence of various abnormal community types driven by opportunistic pathogens (Fig. 1).

**Fig. 2:**
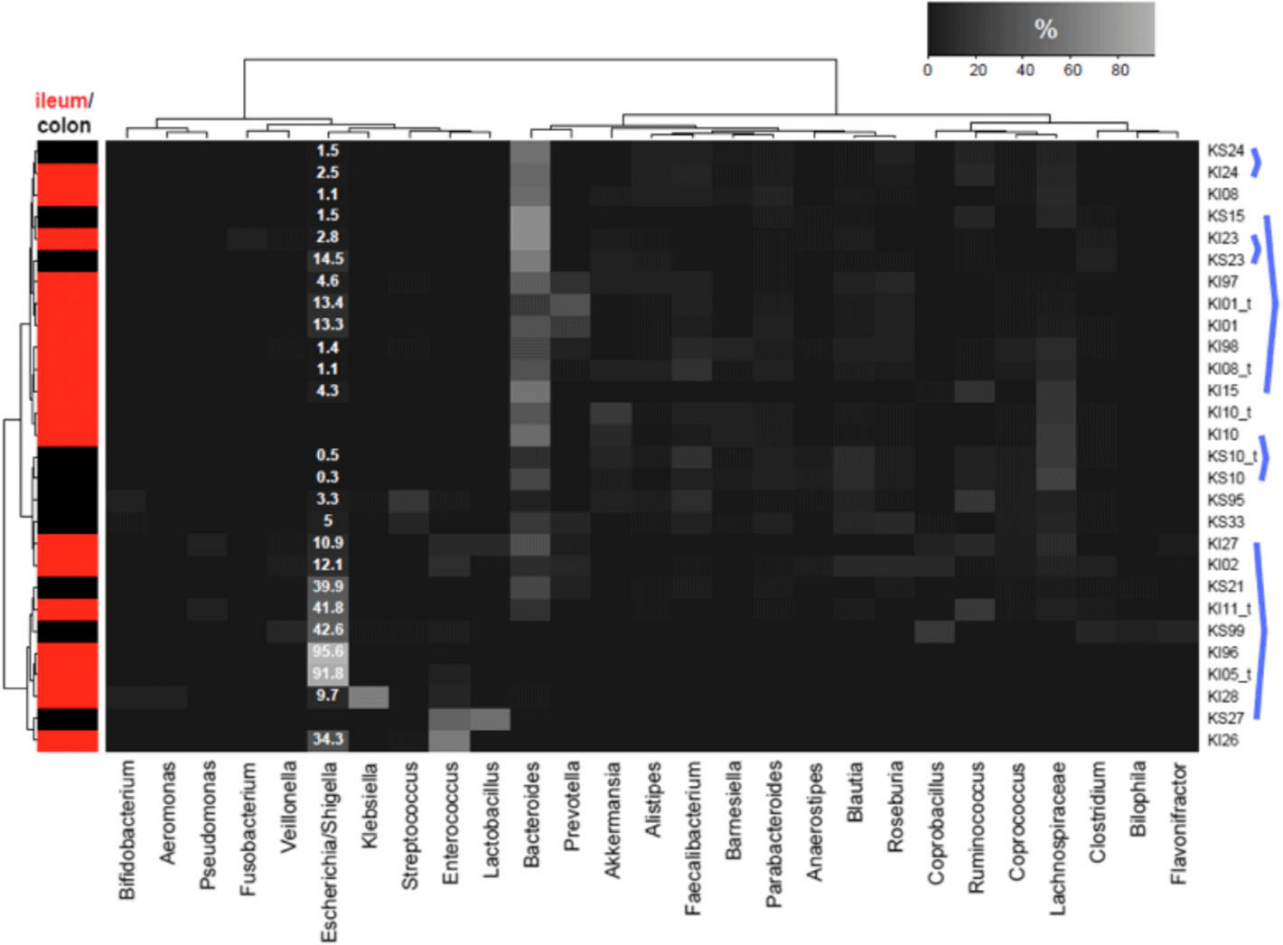
Taxonomic composition of gut metagenomes in Crohn’s disease patients is characterized by the pronounced presence of *Escherichia/Shigella*. The heatmap shows relative abundance of microbial genera (columns) in microbiota samples (rows). The genus levels are provided in percentages of the total bacterial abundance. The blue lines connect pairs of stool and ileal metagenomes from the same patients. Hierarchical clustering is performed using whole-genome UniFrac metric for rows and (1 - Spearman correlation) - for columns; linkage was performed by Ward’s method. Only the major genera (> 3% of the total abundance in at least one sample) are shown

The comparison of the stool metagenome compositions with those of a healthy Russian population as an external control group (*n* = 96) [24] showed a significant shift in the levels of many bacterial species, including a marked enrichment in opportunistic pathogens and decrease in commensal flora (see Fig. 2; a complete list of the significantly over- and underrepresented species is provided in Additional file 1: Table S1). Moreover, the CD metagenomes are dominated by pathogens that were undetected or had a relative abundance close to zero in most healthy subjects (the central observations shown in Fig. 2 are listed below). There is an abnormal domination of Proteobacteria (*Escherichia*, *Enterobacter*, *Pseudomonas* and *Klebsiella*). The major drivers included *Enterococcus faecalis*, *faecium* and *casseliflavus,* which are pathobionts implicated in nosocomial infections that are able to survive oxidative stress and are associated with inflammation [25, 26]. The observed dominance of the species listed is consistent with existing data regarding the CD gut microbiota [7, 14, 22]. In one of the ileal samples, we identified an unexpected presence of *Aeromonas hydrophila* and *veronii* (5%; see Fig. 2); infection with this enteropathogen is associated with a poor clinical outcome in IBD [27]. In another patient, both the stool and ileal samples contained a high fraction (0.4 and 4.2%) of *Fusobacterium varium,* which is a species associated with and able to induce ulcerative colitis [28]. In one ileal sample and one stool sample, we detected a significant fraction of *Clostridium difficile* (3.2 and 7.2%, respectively). Other opportunist pathogens overrepresented in some CD metagenomes included *Clostridium nexile* and *Clostridium clostridioforme* [29]; *Veillonella* and *Flavonifractor*; the sulfate-reducing bacterium *Bilophila wadsworthia*, which is a pathogen associated with appendicitis and other intra-dominal inflammatory disorders [30]; and *Streptococcus infantarius,* which is associated with colon cancer [31]. Some of the CD were enriched in *Ruminococcus gnavus,* which is among the few known representatives of the genus that consumes mucosa and is linked to IBD [32]. The increased levels of *Lactobacillus rhamnosus* (in both stool and ileal samples from one patient, 53 and 8%) and *Bifidobacterium breve* (1 stool sample, 4%) were likely due to the intake of probiotic or dairy products; however, certain studies have shown an increase in the respective genera in microbiota from IBD patients [33, 34] and, for *B. breve*, from patients with alcohol-induced liver cirrhosis [35]. Notably, as seen from Fig. 2, CD metagenomes with increased levels of *Bacteroides* were more prevalent than those with *Prevotella* (13 vs. 1 metagenomes with a genus abundance > 30%). Levels of *Faecalibacterium prausnitzii* were also significantly decreased compared with that in the healthy Russian population (4.0% ± 5.1% vs. 8.5% ± 8.5%, Mann-Whitney test, adjusted *p* = 0.042). This microorganism is recognized as an inhibitor of inflammation in the gastrointestinal tract [36, 37]. Multiple species from *Roseburia*, *Coprococcus*, *Eubacterium* and other related genera from the Firmicutes phylum known to be important gut butyrate-producers were also significantly decreased (Fig. 2).

### Metagenomic analysis of *E. coli* genome suggests the existence of multiple strains within the same patients

One of the most obvious differences between the CD patients and the healthy populations from Russia and other countries was the order of magnitude increase in the *Escherichia coli* relative abundance in the stool samples (2.4% ± 16.5% vs. 0.2% ± 7.7%, one-tailed Mann-Whitney test *p* = 0.00167). The ileal *E. coli* levels were also high (2.8% ± 4.1%) but were not correlated with the respective stool levels in the same patients (*n* = 5, Pearson correlation *r* = − 0.22). Previously, we performed a cultivation-dependent study of this species associated with CD via the isolation and genome sequencing of a subset of the same microbiota samples [38]. To deepen our understanding of the specifics of CD-associated *E. coli*, here, we compared the gene content of this bacterial species reconstructed from “shotgun” metagenomic data with genomes isolated from gut microbiota samples from the same patients (Additional file 1: Table S2).

Overall, the analysis of the metagenomes confirmed the presence of the species with genomes highly similar to that of the respective isolates. The mapping of the metagenomic reads to the corresponding *E. coli* isolate genomes and analysis of the read coverage using the maximum expected coverage (MEC) algorithm (see Materials and methods) showed that for each of the seven metagenomes analyzed, the corresponding *E. coli* isolate genome was the most similar and that the similarity was significantly higher than the similarity between random “genome-metagenome” pairs (MEC = 0.99 ± 0.01 vs. 0.81 ± 0.08, one-tailed Mann-Whitney test, *p* ≤ 10^− 7^) (Additional file 1: Figure S1).

The metagenomic data also supported certain important extra-chromosomal specifics of *E. coli* identified via the genomic analysis of the isolates. The mapping of the metagenomic reads to the sequence of the plasmid pLF82 (present in the CD-associated *E. coli* strain LF82 [23]) yielded 4 samples with a high coverage (17–73% of the plasmid sequence was covered), and all four of the corresponding *E. coli* isolate genomes were shown to have sequences with a high level of identity to pLF82 [38] (Additional file 1: Table S3A). Moreover, some subjects from the healthy Russian population also had metagenomic sequences with identity to this plasmid (Additional file 1: Table S3B); interestingly, these samples had increased fractions of *E. coli* and other opportunistic bacteria (*Streptococcus*, *Enterococcus* and *Klebsiella*).

For a more precise comparison of the genomes and metagenomic profiles of *E. coli* from the same samples, we performed an analysis of metagenomic single-nucleotide polymorphisms (mSNPs) (see Materials and methods). In total, there were 5 “genome-metagenome” pairs in which the metagenome produced sufficient coverage of the *E. coli* genome to perform this analysis. In 3 of these pairs, the distance was orders of magnitude lower than the distance within the mixed “genome-metagenome” pairs (distance d = 0.00026–0.00520 vs. 0.018 ± 0.011, *p* = 0.002, one-tailed Mann-Whitney test; see Additional file 1: Table S4). However, in the other 2 pairs, the distance was significantly higher (d = 0.016 and 0.010). To examine the possibility of subspecies-level diversity in the discrepant pairs, we estimated the mSNP allele frequency. Interestingly, in each of these two pairs, the fraction of mSNPs with a second major allele identical to the letter present in the respective genome was higher than that of any of the mixed “genome-metagenome” pairs involving the same genome (95% vs. 84% ± 4 and 75% vs. 62% ± 5%, respectively). Our results suggest the existence of more than one *E. coli* strain in the microbiota samples and that the sequenced genome corresponds to one of the dominant strains. The analysis of the subspecies-level diversity of the metagenomes using an alternative approach, i.e., the ConStrains tool [39], also revealed the presence of two or more strains of *E. coli* in 7 of the 28 analysed metagenomes (see Additional file 2: Table S5).

### CD-associated *E. coli* is genetically diverse

To assess the gene composition landscape of *E. coli* as reflected by the metagenomic data, we performed pre-mapping to a global gut microbial gene catalogue [40] and selected the genes belonging to *E. coli* from the catalogue. Initially, the *E. coli* pangenome was identified using a representative set of various *E. coli* genomes, including commensal, pathogenic and other strains; then, based on the sequence similarity, the genes corresponding to the *E. coli* pangenome were extracted from the gene catalogue (see Materials and methods). The analysis of the presence/absence of genes was performed on the level of orthology groups (OGs). We separately analysed the part of the pangenome corresponding to an accessory genome (AG) containing 2993 of the 5598 OGs.

Our previous genomic analysis of strains isolated from gut microbiota of CD patients showed that the functions that mostly differentiate CD-associated *E. coli* from commensal strains include the utilization of propanediol (and other sugar alcohols) and iron uptake [38]. We decided to explore how this understanding conforms to the results obtained from the “shotgun” metagenomic profiling of the same and an additional set of samples from CD patients. To analyse the metagenomic profile of the *E. coli* gene content in the context of the available genomic data, we converted a representative set of *E. coli* genomes to an accessory genome presence/absence profile (see Materials and methods) and compared this profile to metagenomic *E. coli* images of CD patients from Russia, the USA [14] and Denmark [10] and healthy populations worldwide. Using the AG profiles, the metagenomic *E. coli* images appeared to be more similar to the genomes of the strains isolated from the same samples (0.25 ± 0.09, *n* = 9 comparisons, binary metric) than all unrelated AG profiles of the worldwide populations were to each other (0.37 ± 0.09, one-tailed Welch t-test, *p* = 0.001); the same result was observed when the comparison was performed using only the *E. coli* virulence genes (17 genes selected as described in the Methods; 0.20 ± 0.28 vs. 0.5 ± 0.19, respectively; *p* = 0.04082). The paired AG profiles of the stool and ileal samples from the same patients were highly similar (d = 0.06 and 0.17, *n* = 2 pairs).

**Fig. 3:**
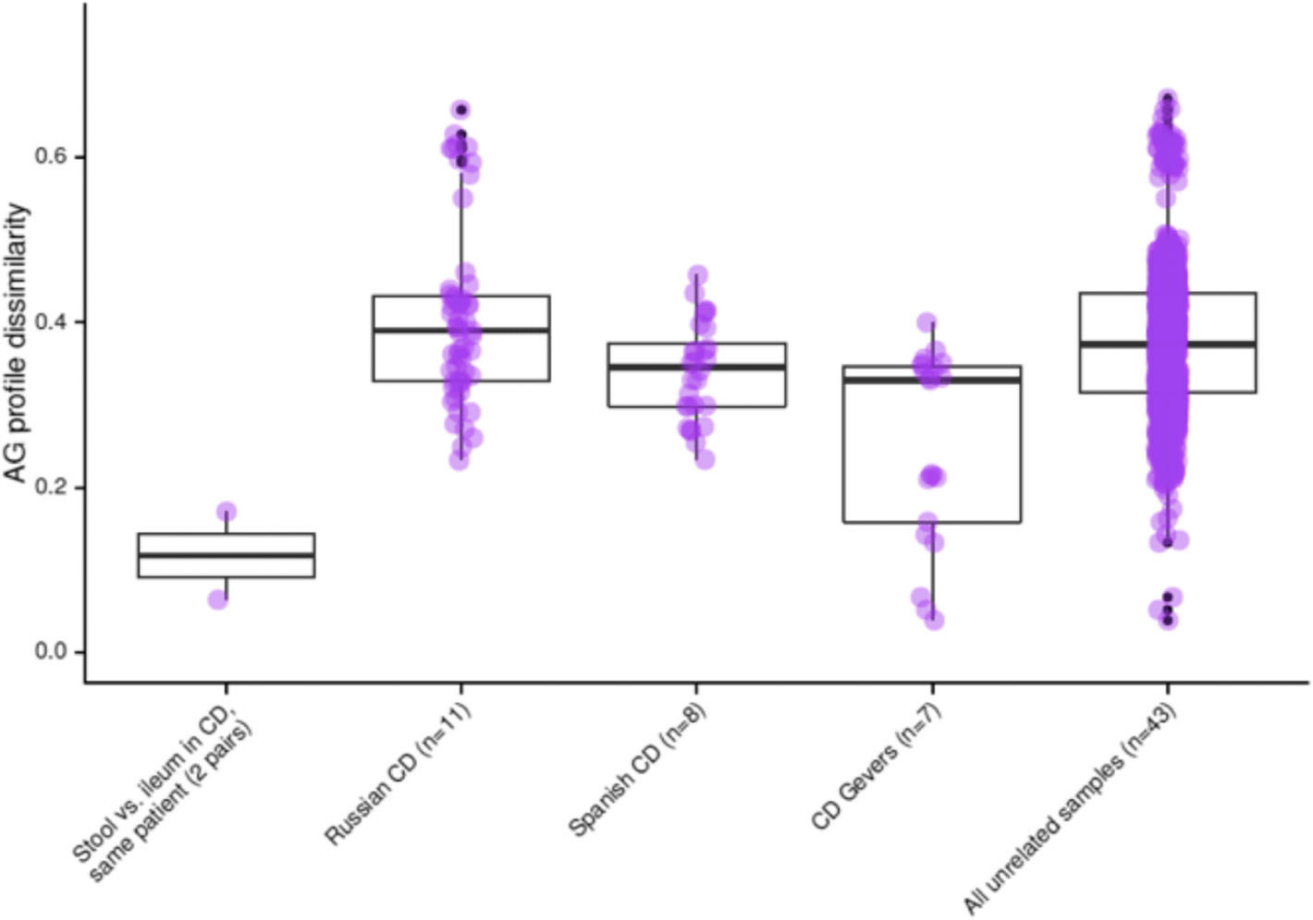
Variability of *E. coli* accessory gene presence profile across different groups of metagenomes. The boxplots show the distributions of pairwise dissimilarity of accessory genome (AG) profiles of *E. coli* calculated for all possible pairs from the following groups of samples: stool and ileal samples from the same Russian CD patient; all Russian CD patients; Spanish CD patients; USA treatment-naive CD patients; as well as the pairs between all unrelated samples. The scatterplots reflect the same information as the boxplots in a more detailed way

Interestingly, the hierarchical cluster analysis of the AG profiles showed a high level of genetic diversity of *E. coli* in the CD metagenomes (Fig. 3) suggesting that CD-associated *E. coli* are not a homogenous group but rather consist of multiple genotypes with diverse genomic repertoires (clustering by the above-mentioned 17 virulence genes also showed diversity, see Additional file 1: Figure S2). The distribution of the dissimilarity values among the AG profiles was comparable to that of the worldwide AG profiles (0.39 ± 0.11, *p* = 0.16; one sample per patient). A similar effect was observed in the Spanish CD patients (0.35 ± 0.06). The USA treatment-naive patients tended to be more similar in their AG profiles (0.33 ± 0.12), which was even more pronounced after removing the single distant sample SAMN02674793 (0.21 ± 0.11).

**Fig. 4:**
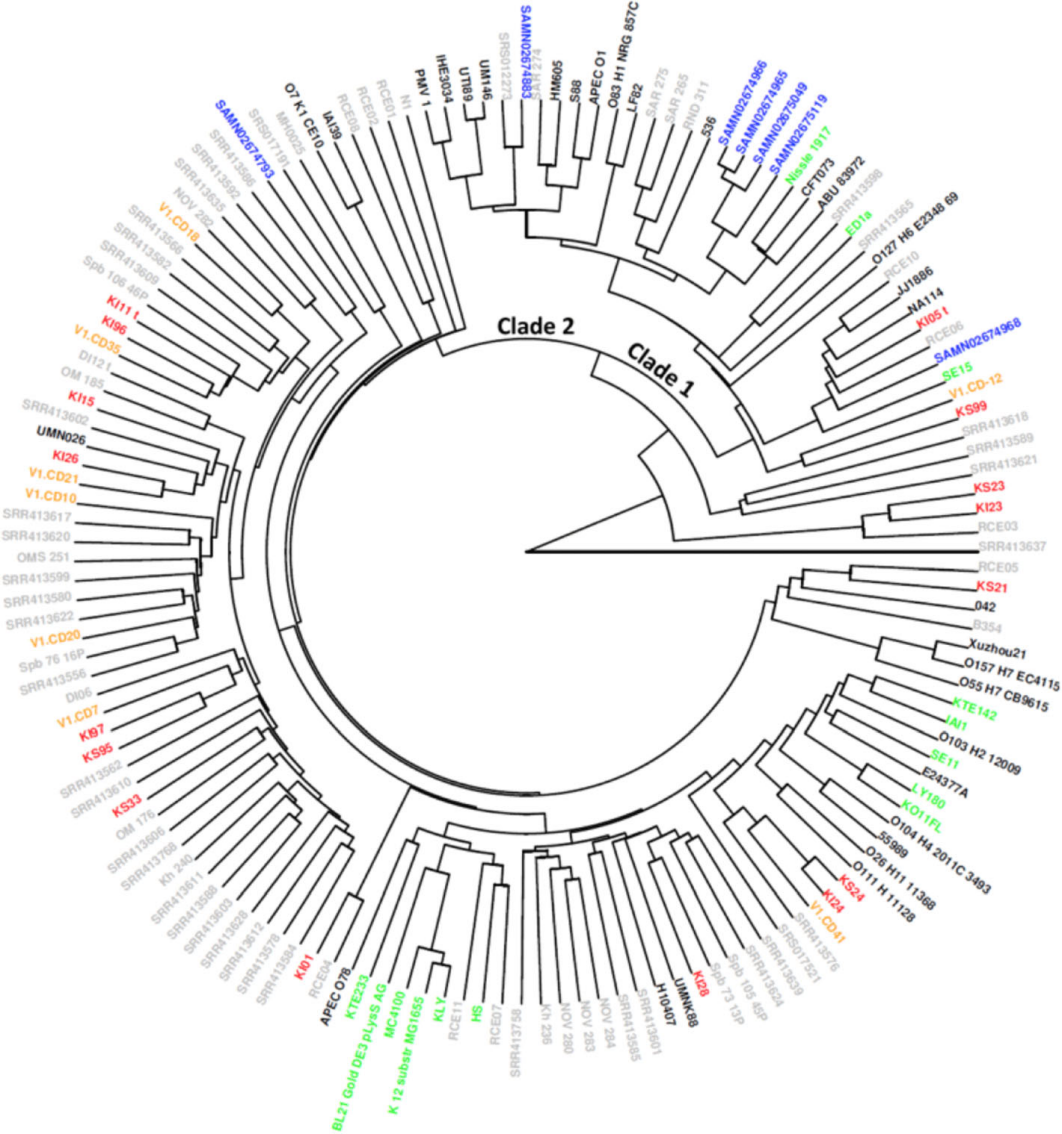
Clustering of the metagenomes and *E. coli* genomes based on a unified representation of the accessory gene presence profile. Clustering is performed by the accessory OG profiles (binary metric, average linkage). Colour legend: black - genomes of pathogenic strains, green - commensal, red - Russian CD, blue - USA treatment-naive CD, orange - Spanish CD (remission), grey - healthy populations and other known *E. coli* genomes (details are in Additional file 1: Table S9)

Except for several strongly outlying samples (CD metagenomes from the same patient KS23 and KI23 with their respective genome RCE03 and three Chinese metagenomes), the tree contained 2 main branches (Clades 1 and 2; see Fig. 4). Regarding the metagenomes from the Russian CD patients, the distribution highly conformed to the results obtained using the multiple alignment of the *E. coli* whole genomes [38]. Clade 1 (including 16 metagenomes and 20 genomes) included all 4 major known CD-associated *E. coli* strains (LF82, O83:H1 [41], UM146 [42] and HM605 [43]) and several other pathogenic strains included in the O6 serogroup. Remarkably, among all CD metagenomes included in the tree, Clade 1 included only 2 of the 13 Russian and only 1 of the 13 Spanish CD metagenomes; however, almost all (6 of 7) of the metagenomes from the treatment-naive USA patients were located in Clade 1.

To identify the genotype of the most recent common ancestor of the pathogen-enriched Clade 1, we identified the genes prevalent in this clade (*n* = 1128; OGs detected in > 80% of the metagenomes) and at the same time rare in Clade 2 (*n* = 798; OGs detected in < 20% of the metagenomes). Forty-seven OGs were obtained. Furthermore, we refined the list by excluding the OGs with a similar OG detected in most Clade 2 members (see Materials and methods), ultimately yielding 34 genes.

The list representing the signature of Clade 1 (Additional file 1: Table S6) contains several genes that are remarkable from the pathobiont arsenal perspective. The proteins coded by some of these genes are known to play important roles in biofilm formation as follows: holin-like protein CidA facilitates the release of DNA that fortifies the biofilm [44], and toxin-antitoxin biofilm protein TabA influences biofilm dispersal [45]. Putative neuraminidase (sialidase) is another gene involved in mucus degradation that potentially contributes to the mucus adherence of *E. coli* as follows: sialidases are known factors involved in the pathogenesis of microbial infections by helping bacteria survive in the outer mucosa layer by cleaving mucin glycoproteins [46, 47]. The levels of its particular type, intramolecular *trans*-sialidase, have been shown to increase in microbiota of IBD patients [48]. Several genes are associated with cell adhesion and fimbriae facilitating successful colonization of the gut, including long polar fimbrial protein LpfD and putative fimbrial-like protein YcbV precursor. The presence of the metallo-beta-lactamase superfamily proteins suggests that resistance to clinically important antibiotics is prevalent among the members of Clade 1. Finally, the presence of microcin-E7 immunity protein (conferring immunity to toxin colicin E7 produced by bacteria) suggests that such a toxin that is normally carried on a plasmid is also prevalent in CD-associated *E. coli* belonging to this clade.

For further validation, a cluster analysis of the reconstructed *E. coli* gene content was additionally performed using the gene-family profiles obtained using an alternative approach, i.e., PanPhlAn [49]. Once the samples from the Russian CD patients were clustered, a tree visually similar to that produced using our approach was obtained (Additional file 1: Figure S3A); this similarity was confirmed by comparing the distance matrices yielded using our method and PanPhlAn (Mantel test *R* = 0.928, *p* = 0.001). Once this set was extended (as an example) by 6 metagenomes from USA treatment-naive CD patients and 7 metagenomes from the Chinese control group, the resulting tree also included 2 main branches that were generally similar to those in the tree based on the AG profiles (Additional file 1: Figure S3B; Mantel test *R* = 0.8824, *p* = 0.001).

### Comparison of accessory genomes to matched controls shows the lack of genetic commonalities in CD-associated *E. coli*

Remarkably, most Russian CD metagenomes were located in Clade 2 (36 genomes and 68 metagenomes; see Fig. 4). Clade 2 contains few pathogenic (or disease-associated) strains. Therefore, we analysed the contents of Clade 2 in another way. *E. coli* in each particular case of CD can possess a specific set of genes that allow it to thrive in the gut environment and produce inflammation. To examine this question in greater detail, we compared the AG profile of each CD metagenome in Clade 2 (11 Russian and 8 non-Russian patients) with the closest match to the metagenome of a healthy subject. While most subjects were not examined endoscopically, at the time of sampling, the subjects did not manifest signs of intestinal discomfort.

Nineteen matched control comparisons were performed. The matched controls closest to CD in terms of the AG profile were the healthy Russian (*n* = 10 comparisons) and Chinese (*n* = 9) populations; the samples within the compared pairs were 1.4 times closer than random pairs (0.23 ± 0.03 vs. 0.33 ± 0.05, *p* = 2.268E-14). In each comparison, 227 ± 76 genes per comparison were identified to be CD-specific (present in CD but lacking in the non-CD control; 1704 unique genes in all comparisons in total). This distribution was found to be generally comparable with that obtained in the inverse comparison (261 ± 165 genes per comparison present in the *E. coli* profile of a control but not in that of a matching CD patient; paired Mann-Whitney test *p* = 0.52; *n* = 1763 unique genes in total).

We analysed the genes that are commonly present in *E. coli* in CD patients but lacking from one of the matched controls (identified in ≥5 of the 19 comparisons). In total, 175 and 205 genes were identified in the CD and control metagenomes, respectively (80 and 137 after discarding the genes with unknown functions, see Additional file 1: Table S7). The lists of CD- and control-specific genes contained a similar fraction of genes related to bacteriophages (15% vs. 19%) and horizontal gene transfer (2.5% vs. 3.6%). No genes related to biofilm formation were included in the lists. However, the CD-specific list was enriched in mercury metabolism genes (5% vs 0%). The *E. coli* accessory genome diversity results showed that most CD metagenomes harbouring *E. coli* strains are genomically diverse and distant from the AIEC pathotype typically described as CD-associated (contained in Clade 1).

Overall, the increasing incidence of inflammatory bowel diseases, including Crohn’s disease, in developed countries is a frightening prospect in terms of morbidity and a challenge for the modern global healthcare system. A meta-analysis showed that the annual incidence is as high as 12.7 and 20.2 per 100,000 person-years in Europe and North America, respectively [50]. The Western lifestyle includes not only the acquisition of material wealth and social security but also the following undesirable additions: underexposure to bacteria during birth and at an early age, the consumption of industrial foods rich in preservatives and artificial additives, a sedentary lifestyle, and decreased intestinal motility. A plethora of these previously underestimated factors contributes to the imbalance of the “host-microbiota” system and is associated with an increased risk of IBD. Given the rapid globalization, Crohn’s disease is a potential threat for all individuals.

To date, studies attempting to identify the ultimate causative factors of this disease have not been successful. In particular, although microbiota may be involved in the pathogenesis [51], its participation is not completely elucidated. The available results suggest that no single reference healthy gut microbiota exists [52]. The species-level composition is known to vary widely but still provides functional homeostasis [53]. We suggest that following external perturbations, the emerging pathological process might easily resolve in some subjects, but in other subjects this process may transform into chronic inflammation and may be due to an imbalance in the microbiota [54]. An important question is where to search for markers of this imbalance, i.e., at the global community, subcommunities or individual opportunist pathogen levels. Several studies have shown associations between particular gut bacterial species and Crohn’s disease. Some of these species have been hypothesized to be causative agents of the disease [17, 55]. We approached this problem from different scales of the gut system, i.e., at the single species level, particularly *E. coli,* which is among the most referenced species in this context [56–58], and the whole gut community level.

Although *E. coli* is the first and most well-studied human gut species, its levels are normally relatively low compared with species that are members of the dominant phyla (Bacteroidetes, Firmicutes and Actinobacteria). The low levels are likely linked to the fact that *E. coli* has a specific ecological niche as follows: this species does not metabolize polysaccharides [59], which is the main source of energy in the colon. While Proteobacteria may normally prevail in the neonate gut to prepare the niche for the further colonization of commensals [58], in adults, the increased abundance of *E. coli* is associated with various diseases, including colon cancer [60], liver diseases [61], cystic fibrosis [62], and alcoholic dependence syndrome [35] probably due to the inflammation that supports the growth of *E. coli* via the selective suppression of commensal taxa [63]; this increase can also occur following treatment with proton pump inhibitors [64].

In our analysis of the gut communities in patients with Crohn’s disease, we revealed significant dysbiosis in stool and ileal metagenomes. One of the most abundant genera was *Bacteroides*. The increase of *Bacteroides* has been associated with the risk for the development of intestinal inflammation [65]. In general, among all commensals, *Bacteroides fragilis* had the highest abundance. Enterotoxigenic *B. fragilis* which possess the *bft* gene has been hypothesized to be associated with IBD [66]; however, the two *bft* gene sequences from the gene catalogue were not detected in any of the CD metagenomes (data not shown). The observed increase in opportunistic pathogens is consistent with previously published results [8, 14]. Interestingly, the disruption of the community structure appears to be due to an increase in different opportunist species in each patient and, thus, lacks a single direction. However, the significantly increased fraction of *E. coli* in comparison with that in the healthy populations from Russia and other countries was a universal feature. The identification of the specific gene content of CD-associated *E. coli* could enhance our understanding of the gene determinants of the active role of the species in inflammation and, based on variability, differentiate possible scenarios related to pathogenesis. We previously demonstrated the broad variability of CD-associated *E. coli* using genomic sequencing of isolates of gut microbiota from patients [38]. Here, we extended the scope of the analysis by approximating the consensus composition of the accessory genome of the species using metagenomic data. Our approach allowed us to avoid cultivation-associated biases and include the extensive volume of published human metagenomic datasets in a comparative analysis.

CD-associated *E. coli* has been previously suggested to be predominantly represented by AIEC strains, including LF82 and O83:H1 [67]. However, our results show that a large number of *E. coli* genotypes were detected in CD patient metagenomes. Genotypes similar to those of the AIEC strains were only detected in a small fraction of CD patients from the Russian and other populations worldwide. The respective Clade 1 *E. coli* strains from CD patient metagenomes possessed genes associated with biofilm formation, antibiotic resistance, mucus and cell adhesion. The presence of genes associated with virulence does not unambiguously imply the pathogenicity of a strain [68, 69]. Thus, a pathogenicity score based on the number of such genes in a genome is unreasonable. However, the genes identified can be considered genetic determinants conferring a high level of fitness to CD-associated strains in the human gut (based on their high abundance). This hypothesis is supported by the fact that in addition to AIEC and several other pathogenic strains, the probiotic strain Nissle 1917 is included in Clade 1. Most CD-associated *E. coli* were found to be located in the Clade 2. An alternative approach for identifying genetic commonalities among *E. coli* in this clade, i.e., comparing the AG profiles with those of matched healthy subjects, only yielded an enrichment in mercury metabolism genes. As heavy metal resistance genes are often carried on plasmids along with antibiotic resistance and virulence factors [70, 71], we speculate that their prevalence is linked to the higher prevalence of mobile elements (particularly plasmids) in CD-associated *E. coli* than in *E. coli* inhabiting the gut of healthy subjects. Interestingly, the richness of the gene repertoire in CD-associated *E. coli* was comparable to those that are in non-CD subjects, although it is generally accepted that pathogenic strains of *E. coli* tend to possess larger genomes than commensal strains [72]. The lack of specific virulence-associated genes commonly distinguishing CD-associated *E. coli* in Clade 2 from the matched control *E. coli* suggests that there are no universal genetic determinants conferring Clade 2 *E. coli* virulence in CD. Generally, our observations suggest that CD-associated *E. coli* do not possess a specifically defined gene composition but is represented by a broad range of biotypes varying in gene content; the species is likely involved in disease progression rather than onset. These results are consistent with a recent cultivation-based study investigating AIEC [73] that failed to identify a molecular property exclusive to this phenotype. Our conclusions support the concept that Crohn’s disease is a syndrome, i.e., a disease in which similar manifestations in multiple cases are caused by different factors in each case. It is possible that some genes detected in our prediction of the *E. coli* accessory gene content from the metagenomic data in fact originated from other species (i.e., had been obtained from *Escherichia/Shigella* spp. via horizontal transfer). However, the general validity of our method of assessing the *E. coli* accessory genome content from the metagenome is supported by the high similarity with the core genomic data of the isolates from the same microbiota samples.

Furthermore, our results extended the previous findings as follows: at a subtler level, the analysis of the *E. coli* subspecies-level diversity within a microbiota of CD patients performed using our metagenomic SNP profiling algorithm and the ConStrains algorithm revealed that some CD patients host multiple strains of *E. coli* in their gut microbiota. Although the data was derived from only a few samples, the considerable size of the effect is supported by the fact that in each of the samples, we identified thousands of sufficiently covered mixed-type mSNPs supporting the heterogeneity. The effect should be further examined via the extensive isolation of multiple strains using a variety of growth media and genome sequencing. Such diversity might reflect microevolutionary snapshots of *E. coli* or its co-existence at different loci in the gut. Previous genomic analyses of isolates demonstrated the possibility that multiple strains of the same species co-exist within the microbiota of the same subjects, such as*, Helicobacter pylori* in the stomach [74, 75] and *Propionibacterium acnes* on the skin [76]. The metagenomics data also provided evidence for such a phenomenon in the microbiota of healthy subjects [39]. Here, for the first time, we demonstrated the genomic heterogeneity of *E. coli* within the same CD patients. This understanding is eye-opening for clinicians because it implies the need to carefully revise the treatment for Crohn’s disease as follows: an antimicrobial therapy scheme targeted against one biotype of *E. coli* might be ineffective against the other biotypes associated with the condition and may cause collateral damage to both the commensal microbiota and organism of a patient. In particular, there is a need to use a diverse set of organisms as part of probiotic treatment scheme as the experimental evidence shows that multiple *E. coli* pathotypes occupy significantly different ecological niches [77].

## Conclusions

Our results expand the current understanding of how microbial components contribute to Crohn’s disease onset and progression and pave the way for the development of better strategies for personalized diagnostics and treatment of inflammatory bowel diseases.

## Methods

### Sample collection

The samples were collected from a cohort of patients with CD (*n* = 19) enrolled at two clinical centres (Moscow Clinical Scientific Center and State Scientific Center of Coloproctology, Moscow, Russian Federation). The inclusion criteria were as follows: patients aged 18 years or older who were endoscopically and radiologically diagnosed with and histologically confirmed to have Crohn’s disease. The exclusion criteria included signs of indeterminate colitis, infectious diseases, anamnesis of total colectomy, the presence of stoma, and antibiotic treatment. The faecal samples were collected prior to the preparation for endoscopy. The bowel preparation was performed with a polyethylene glycol solution. The patients underwent ileocolonoscopy at the clinical centres. During this procedure, the ileum liquid content was aspirated. In total, 9 stool and 15 ileal samples were obtained (Additional file 1: Table S8).

### Sample preparation protocol

The stool samples were prepared according to the following protocol. A portion of 0.3–0.5 g of faeces was added to 10 ml of PBS, vigorously vortexed for 2–5 min and repeatedly cooled on ice. Human cells and debris were pelleted by centrifugation for 10 min at 0.1 g. The supernatants were collected and placed on ice. The resuspension and centrifugation of the pellet were repeated 3–5 times. The combined supernatant was centrifuged for 20 min at 13.5 g to collect the microbial fraction. The pellet was resuspended, washed twice with PBS and stored at − 20 °C.

The ileal samples were collected as ileal lavage fluid collected during endoscopy by washing the ileal wall with saline. In total, 1 ml of fluid was used for the DNA extraction from such samples. Unlike the stool sample preparation, a lower dilution (3–5 times) and a lower number of repeats of resuspension and centrifugation (no more than twice) were performed.

### Preparation of metagenomic libraries and “shotgun” sequencing

The “shotgun” libraries were prepared for the sequencing platforms SOLiD 4, SOLiD 5500 and Ion Torrent (Life Technologies, USA). The sequencing was performed according to the manufacturer’s instructions. For SOLiD 4, the SOLiD Fragment Library Construction Kit, SOLiD Fragment Library Barcoding Module 1–16, SOLiD EZ Bead TM E80 System Consumables, SOLiD ToP Sequencing Kit and MM50/5 (Life Technologies, USA) were used. For SOLiD 5500, the 5500 SOLiD Fragment Library Core Kit, SOLiD Fragment Library Barcoding Kit, SOLiD FlowChip Kit, SOLiD FWD SR S50 Kit and SOLiD Run Cycle Buffer Kit (Life Technologies, USA) were used. For Ion Torrent PGM, the Ion Xpress Plus Fragment Library Kit, Ion Sequencing Kit, Ion PGM Template OT2 200 Kit, Ion PGM Sequencing 200 Kit and Ion 318 Chip Kit (Life Technologies, USA) were used.

Three of the samples were sequenced using more than one platform; in total, 28 metagenomic read sets were obtained (Additional file 1: Table S8).

### Taxonomic and functional profiling of metagenomes

All analytical steps of the study are summarized in the Additional file 1: Figure S4. The taxonomic composition analysis was performed by mapping the metagenomic reads against a representative non-redundant reference catalogue of 353 gut microbial genomes [78] and estimating the relative abundance levels based on the obtained coverage profiles as previously described [79]. The functional composition was assessed in a similar way as previously described by using a reference gene catalogue containing 3.3 mln gut microbial genes [40]. For the comparative analysis, stool metagenomic datasets from 3 studies involving the following healthy populations worldwide were used: China (*n* = 68) [80], Denmark (*n* = 85) [40], and USA (*n* = 138) [81]; in addition, Crohn’s disease patients from the USA (treatment-naive paediatric patients; *n* = 17) [14] and Spain (clinical remission, n = 13) [10, 40] were included.

### Analysis of maximum-expected coverage

The bacterial species present in a microbiota sample might possess genome(s) that considerably differ from the reference genome available as a representative of this species. In such cases, even if the sequencing depth was infinitely increased, the covered part of the genome could converge to a value less than 100%; the higher the value, the more representative the reference genome. To assess this limit, we assumed that the non-covered part of the genome exponentially decreases as the coverage depth increases and introduced a maximum expected coverage (MEC) value as follows:

$$ \alpha =\frac{f}{1-\exp \left(-\frac{\mathrm{NL}}{G}\right)} $$

where *N* is the number of mapped reads, *L* is the read length, *G* is the genome size, and *f* is the number of positions in a genome that were covered by at least 1 read. The MEC is an indirect measure of the similarity of the gene content between the reference genome and the bacterial species present in the analysed sample.

### Analysis of metagenomic SNPs

To assess the subspecies level genomic variability of *E. coli* using “shotgun” metagenomes, an algorithm for metagenomic SNP (mSNP) calling was developed. This approach is similar to SNP calling for a single sequenced genome and uses heuristically selected parameters. Essentially, the mapping of a single metagenomic readset to a reference genome catalogue yields an alignment file (in BAM format) containing the coverage profile of each of the genomes. In sufficiently covered genomes (with MEC ≥ 0.5 and mean coverage ≥5×), only the nucleotide positions with coverage ≥4× are considered. For each such position, a consensus letter is determined using ad hoc scripts as a letter supported by the highest number of reads (which should not be less than 4); if two major alleles are supported by an identical number of reads, one allele is randomly selected. A metagenomic SNP is defined as a position in a consensus sequence with an alternate letter compared to the reference genome. As a genomic dissimilarity measure of *E. coli* species between two metagenomes, we measured the Hamming distance between the two respective consensus sequences adjusted based on the coverage information; only the genome fragments belonging to core genes were used. Specifically during this analysis focused on metagenomic SNPs, each metagenome was mapped to a reference genome of the *E. coli* strain K-12 alone. To compare the *E. coli* genomes with the metagenomic profiles of *E. coli*, each isolated genome was randomly fragmented into reads (mean targeted coverage 100×) before being subjected to identical consensus sequence identification (mapping to a reference genome catalogue, etc.).

### Selection of *E. coli* pangenome and accessory genes in the global gene catalogue

A representative set of 81 diverse *E. coli* genomes was formed (among them, 28 genomes belonged to commensal strains and 43 genomes belonged to pathogenic strains, including 4 known CD-associated strains [23, 42, 43, 82] and 10 strains previously isolated from Russian CD patients from the examined cohort [38]) (Additional file 1: Table S9). Using this set, gene orthology groups (OGs) were constructed using OrthoMCL [83] by 50% identity of amino acid sequences. As some of the produced OGs were divided by the clustering algorithm into multiple groups due to the incompleteness of the gene sequences, to compensate for this effect, the potentially divided groups were merged using the following heuristic: for each two OGs A and B, pairwise BLASTp alignments of a gene from A to a gene from B were considered for such possible pairs; the score S = (% identity) × (% match) was computed for each pair; if the average S across all gene pairs exceeded 6400 (80 × 80), the ОGs were merged into a single OG.

The nucleotide sequences of all genes of the obtained OGs were aligned against the 3.3 mln gut microbial reference gene catalogue to yield highly similar matches (similarity criterion: > 80% identity and > 80% of both subject and query sequence lengths). The obtained 9125 matching genes in the catalogue belonged to 5598 OGs; these genes were further used as a template to assess the presence of the *E. coli* pangenome in the gut metagenomes (the detailed statistics used for the pangenome construction are described in Additional file 1: Table S10). Core genes (genes occurring in all 81 genomes) were subtracted from the pangenome to yield a set of *E. coli* accessory genes (AG) (2993 OGs).

Virulence-associated genes were identified among the accessory genes of *E. coli* by aligning the sequences of the latter to a published list of 76 *E. coli* virulence-associated genes [84] (BLASTn search was performed with the following thresholds: e-value <1E-5, sequence coverage > 80%, identity > 80%). The search yielded 17 genes listed in the Additional file 1: Table S11.

### Profiling of *E. coli* gene content in metagenomes and genomes

The gene content of *E. coli* in the gut metagenomes was estimated in the form of a binary vector of the presence/absence of each gene included in the pangenome of the species. A gene was considered present if at least 1 read was mapped to the gene during the mapping of all reads to the reference gene catalogue. Here, to adjust for variation in the sequencing coverage and *E. coli* relative abundance across the metagenomes, for each metagenome, a random subsampling was simulated such that the total number of reads mapped to the pangenome was 80,000. Metagenomes with a lower number of pangenomic reads or less than 50% coverage of the pangenome were not considered in the analysis of the pangenome and accessory genome. The accessory gene (AG) profile was obtained from the pangenome presence profile by filtering genes corresponding to the core genome. A pangenome and accessory profiles were also produced for genomes in the same format based on the alignment of the genomes against the reference gene catalogue [40] using BLASTn (similarity criterion: > 80% identity for > 80% of the gene length). The pairwise dissimilarity between the AG profiles was calculated using a binary metric (using the function dist in R package stats). Hierarchical clustering was performed using the average method.

During the stage of refining the orthology groups specific to Clade 1, the candidate signature OGs of Clade 1 with an OG with an identical function description detected in ≥20% of the AG profiles in Clade 2 were excluded. Here, the OG similarity score was defined as a product of the percent sequence identity and percent query matching length averaged over all possible pairs of genes between the two groups.

### Alternative profiling of *E. coli* gene content

To validate the results of the clustering based on the AG profiles, we used PanPhlAn tool [49], which maps the metagenomic reads against the reference genomes of the target species and then, based on the gene coverage levels, reconstructs the unique gene set of a strain present in the sample. The resulting gene content of *E. coli* in the metagenome is also in the form of a binary vector of the presence/absence of each gene family. The pre-processed database of the *E. coli* pangenome contained 34,881 gene families. The threshold for a gene family to be considered present in the final presence/absence gene profile was set to a coverage depth > 0.5× times the mean coverage.

## Additional files

Additional file 1: Additional Tables and Figures. (ZIP 816 kb)

Additional file 2: Results of subspecies-level analysis of *E. coli* and other microbes in the metagenomes using ConStrains. The columns include: 1 - species name, 2 - number of the detected strain (equal to "NA,insufficient" if no multiple strains were detected), 3 - number of masked samples (equal to 1 if read coverage was insufficient for strain detection), 4 - relative abundance of the strain. (XLSX 167 kb)

## Abbreviations

AG: Accessory genome
AIEC: Adherent-invasive *Escherichia coli*
CD: Crohn’s disease
IBD: Inflammatory bowel diseases
MEC: Maximum-expected coverage
mSNP: Metagenomic single-nucleotide polymorphism
OG: Orthology group
PCDAI: Pediatric Crohn’s Disease Activity Index
