# Supplementary material for: Genetic diversity of Escherichia coli in gut microbiota of patients with Crohn’s disease discovered using metagenomic and genomic analyses
Source: BMC Genomics. 2018 Dec 27;19:968. doi: 10.1186/s12864-018-5306-5 (PMC6307143; doi:10.1186/s12864-018-5306-5)
Supplement: Supplementary file 1 — Additional Tables and Figures. (ZIP 816 kb) [file 12864_2018_5306_MOESM1_ESM.zip › Additional_Data.docx]

# Additional Data

## Additional tables

**Additional Table 1 - Bacterial taxa significantly over-/underrepresented in stool metagenomes of Crohn’s disease patients (n=9) in comparison with the control group (n=96). A) Genera; B) species (according to the names of reference genomes). In the last column, “Inf” value corresponds to the case when the median for the first group was positive, while for the second group it was zero; “NaN” value corresponds to the case when the medians for the both groups were zero.**

**A)**

| **Taxon** | **FDR adj. p-value** | **Median, group 1** | **SD, group 1** | **Median, group 2** | **SD, group 2** | **Ratio of medians in group 1 and 2** |
| --- | --- | --- | --- | --- | --- | --- |
| **Decreased in CD:** |  |  |  |  |  |  |
| Acidaminococcus | 0.0128 | 0.0625 | 0.2108 | 0 | 0.0264 | Inf |
| Catenibacterium | 0.0101 | 0.2772 | 2.552 | 0 | 0.1274 | Inf |
| Dialister | 0.037 | 0.0831 | 2.9859 | 0 | 0.0504 | Inf |
| Methanobrevibacter | 0.0048 | 0.2278 | 1.9494 | 0 | 0.0542 | Inf |
| Sporacetigenium | 0.0128 | 0.0651 | 0.1836 | 0 | 0.0419 | Inf |
| Bifidobacterium | 0.0351 | 0.4636 | 5.985 | 0.0129 | 1.4602 | 35.96 |
| Prevotella | 0.0101 | 4.3007 | 20.7655 | 0.1733 | 2.9314 | 24.81 |
| Coprococcus | 0.0024 | 7.0111 | 7.1157 | 0.8534 | 1.3171 | 8.22 |
| Eubacterium | 0.0247 | 1.3952 | 1.6135 | 0.2821 | 0.895 | 4.95 |
| Dorea | 0.0048 | 1.17 | 1.7879 | 0.2534 | 0.5658 | 4.62 |
| Ruminococcaceae | 0.0247 | 0.4919 | 0.5006 | 0.1122 | 0.4278 | 4.38 |
| Faecalibacterium | 0.0152 | 8.4955 | 8.469 | 3.1255 | 3.9553 | 2.72 |
| **Increased in CD:** |  |  |  |  |  |  |
| Enterobacter | 0.0058 | 0 | 0.1087 | 0.1586 | 0.2678 | 0 |
| Klebsiella | 0.0013 | 0 | 0.3236 | 0.0826 | 0.566 | 0 |
| Salmonella | 7.00E-04 | 0 | 0.0201 | 0.0213 | 0.0755 | 0 |
| Escherichia/Shigella | 0.023 | 0.1868 | 7.7738 | 3.3422 | 17.127 | 0.06 |
| Aggregatibacter | 0.0085 | 0 | 0 | 0 | 0.0021 | NaN |
| Candida | 0.0085 | 0 | 0 | 0 | 0.0203 | NaN |
| Enterobacteriaceae | 0.0085 | 0 | 0.0146 | 0 | 0.0561 | NaN |
| Mycoplasma | 0.0085 | 0 | 0 | 0 | 0.0012 | NaN |
| Pseudomonas | 0.0085 | 0 | 0 | 0 | 0.0112 | NaN |
| Yokenella | 7.00E-04 | 0 | 0.0077 | 0 | 0.0271 | NaN |

**B)**

| **taxon** | **FDR adj. p-value** | **Median, group 1** | **SD, group 1** | **Median, group 2** | **SD, group 2** | **Ratio of medians in group 1 and 2** |
| --- | --- | --- | --- | --- | --- | --- |
| **Decreased in CD:** |  |  |  |  |  |  |
| Acidaminococcus_sp_D21 | 0.0113 | 0.058 | 0.0695 | 0 | 0.0256 | Inf |
| Bifidobacterium_adolescentis_ATCC_15703 | 0.0156 | 0.2863 | 4.6987 | 0 | 0.7946 | Inf |
| Blautia_hydrogenotrophica_DSM_10507 | 0.0171 | 0.0428 | 0.0846 | 0 | 0 | Inf |
| Catenibacterium_mitsuokai_DSM_15897 | 0.0053 | 0.2772 | 2.552 | 0 | 0.1212 | Inf |
| Clostridium_bartlettii_DSM_16795 | 0.0377 | 0.0651 | 0.1836 | 0 | 0.0514 | Inf |
| Methanobrevibacter_smithii_ATCC_35061 | 0.0029 | 0.2278 | 1.9494 | 0 | 0.0514 | Inf |
| Ruminococcus_sp_18P13 | 0.0362 | 0.0758 | 0.5293 | 0 | 0.0965 | Inf |
| Prevotella_copri_DSM_18205 | 0.0022 | 3.9772 | 19.5915 | 0.0197 | 2.279 | 202.35 |
| Coprococcus_eutactus_ATCC_27759 | 0.0013 | 0.6577 | 4.8439 | 0.0668 | 0.0649 | 9.84 |
| Eubacterium_siraeum_70_3 | 0.0493 | 0.2833 | 0.8574 | 0.0324 | 0.2338 | 8.74 |
| Coprococcus_sp_ART55_1 | 0.0013 | 0.5265 | 3.3832 | 0.0725 | 0.1027 | 7.27 |
| Eubacterium_dolichum_DSM_3991 | 0.0053 | 0.1209 | 0.1057 | 0.0175 | 0.0516 | 6.91 |
| Faecalibacterium_prausnitzii_SL3_3 | 0.0178 | 2.7326 | 2.594 | 0.5078 | 1.7129 | 5.38 |
| Butyrivibrio_crossotus_DSM_2876 | 0.0113 | 0.3595 | 5.7342 | 0.0712 | 0.1595 | 5.05 |
| Dorea_longicatena_DSM_13814 | 0.0049 | 1.0551 | 1.7757 | 0.2185 | 0.5147 | 4.83 |
| Clostridium_sp_L2_50 | 0.0049 | 0.492 | 3.832 | 0.1192 | 0.2282 | 4.13 |
| Faecalibacterium_cf_prausnitzii_KLE1255 | 0.0113 | 2.138 | 3.5189 | 0.5308 | 0.8494 | 4.03 |
| Bacteroides_pectinophilus_ATCC_43243 | 0.0148 | 0.562 | 3.4614 | 0.146 | 0.2664 | 3.85 |
| Eubacterium_ventriosum_ATCC_27560 | 0.0113 | 0.3432 | 0.6188 | 0.1015 | 0.2198 | 3.38 |
| Roseburia_intestinalis_XB6B4 | 0.0053 | 0.926 | 1.845 | 0.2759 | 0.3565 | 3.36 |
| Coprococcus_catus_GD_7 | 0.0148 | 0.7257 | 0.5632 | 0.2584 | 0.3257 | 2.81 |
| Eubacterium_biforme_DSM_3989 | 0.0483 | 0.2454 | 1.2039 | 0.099 | 0.5727 | 2.48 |
| **Increased in CD:** |  |  |  |  |  |  |
| Bacteroides_clarus_YIT_12056 | 0.045 | 0 | 0.095 | 0.2077 | 0.3901 | 0 |
| Citrobacter_freundii_4_7_47CFAA | 0.0387 | 0 | 0.0183 | 0.0083 | 0.0639 | 0 |
| Citrobacter_koseri_ATCC_BAA_895 | 0.045 | 0 | 0.0555 | 0.0129 | 0.0859 | 0 |
| Enterobacter_asburiae_LF7a | 0.0014 | 0 | 0.01 | 0.0035 | 0.0339 | 0 |
| Enterobacter_cancerogenus_ATCC_35316 | 0.0014 | 0 | 0.0097 | 0.0042 | 0.0299 | 0 |
| Enterobacter_cloacae_SCF1 | 0.0014 | 0 | 0.0134 | 0.0095 | 0.042 | 0 |
| Enterobacter_cloacae_subsp_cloacae_ATCC_13047 | 0.0014 | 0 | 0.0229 | 0.0382 | 0.1085 | 0 |
| Enterobacter_cloacae_subsp_cloacae_NCTC_9394 | 0.0213 | 0 | 0.0685 | 0.0394 | 0.0581 | 0 |
| Klebsiella_pneumoniae_342 | 0.0051 | 0 | 0.3236 | 0.0652 | 0.5435 | 0 |
| Salmonella_enterica_subsp_enterica_serovar_Typhimurium_LT2 | 0.0017 | 0 | 0.0201 | 0.0106 | 0.0735 | 0 |
| Escherichia_coli_str_K12_substr_MG1655 | 0.0218 | 0.0959 | 4.0509 | 1.3548 | 9.7717 | 0.07 |
| Escherichia_sp_1_1_43 | 0.0413 | 0.0698 | 2.8326 | 0.7295 | 3.4764 | 0.1 |
| Aggregatibacter_segnis_ATCC_33393 | 0.0213 | 0 | 0 | 0 | 0.002 | NaN |
| Candida_albicans | 0.0213 | 0 | 0 | 0 | 0.0106 | NaN |
| Candida_dubliniensis_CD36_1 | 0.0213 | 0 | 0 | 0 | 0.0087 | NaN |
| Citrobacter_youngae_ATCC_29220 | 0.0248 | 0 | 0.0146 | 0 | 0.0543 | NaN |
| Enterobacter_sp_638 | 0.0311 | 0 | 0.0084 | 0 | 0.0259 | NaN |
| Fusobacterium_ulcerans_ATCC_49185 | 0.0213 | 0 | 0 | 0 | 0.0134 | NaN |
| Fusobacterium_varium_ATCC_27725 | 0.0213 | 0 | 0 | 0 | 0.1292 | NaN |
| Lactobacillus_rhamnosus_GG | 0.0014 | 0 | 0.0083 | 0 | 16.7669 | NaN |
| Mycoplasma_hominis_ATCC_23114 | 0.0213 | 0 | 0 | 0 | 0.0011 | NaN |
| Pseudomonas_mendocina_ymp | 0.0213 | 0 | 0 | 0 | 0.0106 | NaN |
| Staphylococcus_aureus_subsp_aureus_TW20 | 0.0017 | 0 | 0.0055 | 0 | 0.1074 | NaN |
| Staphylococcus_warneri_L37603_contig00102 | 0.0213 | 0 | 0 | 0 | 0.0523 | NaN |
| Yokenella_regensburgei_ATCC_43003_ | 0.0014 | 0 | 0.0077 | 0 | 0.0263 | NaN |

**Additional Table 2 - Correspondence of CD metagenomes with the isolated genomes from Rakitina et al., 2017.**

| **Genome** | **Metagenomes from the same patient** |
| --- | --- |
| RCE01 | KS15, KI15 |
| RCE02 | KI11_t |
| RCE03 | KS23, KI23 |
| RCE04 | KI01, KI01_t |
| RCE05 | KS21 |
| RCE06 | KI05_t |
| RCE07 | KI02 |
| RCE08 | KI97 |
| RCE10 | KI96 |

**Additional Table 3 - Results of plasmid detection using metagenomic data (mapping to the complete sequence of the plasmid pLF82 with length 108,379 bp). Only the samples for which >0.1% of plasmid genome received coverage are shown.**

A)

| **Metagenome ID** | **Total length of mapped reads** | **Covered fraction of the plasmid sequence, %** |
| --- | --- | --- |
| KI01 | 477312 | 73.63327 |
| KI15 | 1542048 | 62.90794 |
| KI26 | 82272 | 32.99071 |
| KS15 | 25488 | 17.79773 |
| KS99 | 65712 | 2.70163 |
| KI28 | 20448 | 2.581681 |
| KS21 | 17280 | 2.472804 |
| KS24 | 2736 | 2.012383 |
| DC06 | 2352 | 1.70605 |
| DI06 | 2112 | 1.279768 |
| KI27 | 1488 | 1.276078 |
| KI08 | 1248 | 1.120143 |
| DI14 | 1248 | 1.107226 |
| DI07 | 912 | 0.841491 |
| DC19 | 816 | 0.708624 |
| KS10 | 816 | 0.575757 |
| DC07 | 528 | 0.487179 |
| KI10 | 480 | 0.44289 |
| DC14 | 336 | 0.310023 |
| DI19 | 288 | 0.265734 |
| KI02 | 240 | 0.221445 |
| KS27 | 192 | 0.177156 |

B)

| **Metagenome ID** | **Total length of mapped reads** | **Covered fraction of the plasmid sequence, %** |
| --- | --- | --- |
| Spb_73_13P | 39552 | 24.47891 |
| Kh_240 | 26544 | 17.75529 |
| OMS_251 | 16368 | 12.11213 |
| NOV_283 | 16752 | 4.166859 |
| Spb_106_46P | 5040 | 3.923269 |
| NOV_280 | 7584 | 1.807546 |
| Spb_76_16P | 5616 | 1.437548 |
| OM_185 | 7248 | 1.09154 |
| Spb_61_1P | 1248 | 1.08139 |
| Spb_105_45P | 1776 | 0.942064 |
| OM_176 | 2400 | 0.90977 |
| NOV_282 | 960 | 0.547154 |
| TYV_203 | 672 | 0.545309 |
| NOV_284 | 576 | 0.498252 |
| RND_314 | 480 | 0.44289 |
| OM_184 | 432 | 0.398601 |
| RND_313 | 480 | 0.398601 |
| Kh_236 | 528 | 0.372766 |
| OM_179 | 528 | 0.368153 |
| TYV_210 | 384 | 0.332168 |
| RND_311 | 576 | 0.315559 |
| OM_180 | 384 | 0.310023 |
| NOV_287 | 384 | 0.298028 |
| OM_178 | 384 | 0.297106 |
| Kh_232 | 384 | 0.280497 |
| TYV_216 | 432 | 0.276806 |
| SAR_274 | 528 | 0.27127 |
| Spb_70_10P | 288 | 0.265734 |
| TYV_212 | 288 | 0.228827 |
| Kh_245 | 240 | 0.204837 |
| NOV_281 | 240 | 0.178079 |
| SAR_270 | 192 | 0.177156 |
| RND_317 | 192 | 0.159625 |
| SAR_271 | 336 | 0.136558 |
| NOV_286 | 192 | 0.13379 |
| TAT_143 | 144 | 0.132867 |
| Spb_103_43P | 144 | 0.132867 |
| SAR_265 | 144 | 0.132867 |
| SAR_264 | 192 | 0.132867 |
| Spb_72_12P | 144 | 0.110723 |

**Additional Table 4 - Genomic dissimilarity between isolated genomes and metagenomic images of *E. coli* according to mSNP measure. Columns include all genomes, rows - all metagenomes providing sufficient coverage of *E. coli* to perform mSNP calling (see Methods).**

|  | **KI15 (01)** | **KI11_t (02)** | **KS21 (05)** | **KI97 (08)** | **KI05_t (06)** |
| --- | --- | --- | --- | --- | --- |
| **RCE01** | 0.000269 | 0.009212 | 0.008292 | 0.007813 | 0.024377 |
| **RCE02** | 0.010842 | 0.005246 | 0.010895 | 0.010074 | 0.023875 |
| **RCE03** | 0.049723 | 0.045495 | 0.048006 | 0.053058 | 0.047996 |
| **RCE04** | 0.007378 | 0.009172 | 0.008457 | 0.007844 | 0.024269 |
| **RCE05** | 0.020629 | 0.01815 | 0.015976 | 0.020545 | 0.02342 |
| **RCE06** | 0.023871 | 0.019282 | 0.022388 | 0.023754 | 0.000211 |
| **RCE07** | 0.0069 | 0.00905 | 0.008186 | 0.008157 | 0.024287 |
| **RCE08** | 0.011486 | 0.009082 | 0.01157 | 0.010002 | 0.023941 |
| **RCE10** | 0.023805 | 0.019119 | 0.022256 | 0.023562 | 0.000216 |

**Additional Table 5 - Results of subspecies-level analysis of E. coli and other microbes in metagenomes using ConStrains. [provided as a separate file Additional_Table_5.xlsx].**

**Additional Table 6 - Accessory genes prevalent in Clade 1 according to the orthology group presence analysis for genomes and metagenomes of the Clade (7 genes with hypothetical function are not listed).**

| 1 | 1,3-propanediol dehydrogenase |
| --- | --- |
| 2 | 3-hexulose-6-phosphate isomerase |
| 3 | 4-hydroxy-tetrahydrodipicolinate synthase |
| 4 | 4-hydroxythreonine-4-phosphate dehydrogenase 2 |
| 5 | anaerobic benzoate catabolism transcriptional regulator |
| 6 | Ben and cat operon transcriptional regulator |
| 7 | C4-dicarboxylate transport transcriptional regulatory protein DctD |
| 8 | Chaperone protein FimC precursor |
| 9 | Citrate/succinate antiporter |
| 10 | Dihydrolipoyl dehydrogenase |
| 11 | Dipeptide transport system permease protein DppB |
| 12 | EIICBA-Glc |
| 13 | Excisionase-like protein |
| 14 | Holin-like protein CidA |
| 15 | HTH-type transcriptional repressor GlcR |
| 16 | Inner membrane protein YohK |
| 17 | L-lactate dehydrogenase |
| 18 | long polar fimbrial protein LpfD |
| 19 | Metallo-beta-lactamase superfamily protein |
| 20 | Microcin-E2 immunity protein |
| 21 | Microcin-E7 immunity protein |
| 22 | MurPQ operon repressor |
| 23 | NAD(P)H dehydrogenase (quinone) |
| 24 | Nucleoside-specific channel-forming protein, Tsx |
| 25 | polynucleotide kinase |
| 26 | putative DNA-binding transcriptional regulator |
| 27 | putative fimbrial-like protein YcbV precursor |
| 28 | putative neuraminidase (sialidase) |
| 29 | Stage 0 sporulation protein KE |
| 30 | Toxin-antitoxin biofilm protein TabA |
| 31 | transporter, divalent anion:Na+ symporter (DASS) family |
| 32 | type IV/VI secretion system protein, DotU family |
| 33 | type VI secretion lipoprotein, family |
| 34 | type VI secretion-associated protein, family |

**Additional Table 7 - The genes most frequently distinguishing an *E. coli* AG profile of a CD patient from one of its matched control. Hypothetical genes are omitted.**

| Genes frequently detected in CD in comparison with the matched control metagenomes | Genes frequently detected in matched control in comparison with the CD metagenomes |
| --- | --- |
| 3',5'-cyclic adenosine monophosphate phosphodiesterase CpdA  3-oxoacyl-[acyl-carrier-protein] reductase FabG  4-hydroxybenzoyl-CoA reductase subunit beta  Acetylxylan esterase precursor  Acetylxylan esterase precursor  Antitoxin HigA  Arylsulfate sulfotransferase AssT precursor  Ascorbate-specific phosphotransferase enzyme IIA component  Ascorbate-specific phosphotransferase enzyme IIB component  Bacteriophytochrome cph2  baseplate wedge subunit  Beta-galactosidase BglY  Biofilm development protein YmgB/AriR  Catabolite repression HPr  conjugal transfer protein TrbG  conjugal transfer relaxase TraI  conjugal transfer transcriptional regulator TraJ  CRISPR-associated endoribonuclease Cse3  Crossover junction endodeoxyribonuclease RusA  Crossover junction endodeoxyribonuclease RusA  Cytochrome c-type biogenesis protein CcmD  Deoxyribose operon repressor  Distal rod protein  Filamentous hemagglutinin  flagellar assembly protein H  Gluconate utilization system GNT-I transcriptional repressor  gpW  Helix-turn-helix  Hemolysin transporter protein ShlB precursor  Hemolysin, chromosomal  Homocysteine S-methyltransferase  IS1 transposase  Lactose transport system permease protein LacG  Lactose-proton symport  L-fucose permease  L-lactate utilization operon repressor  Macrophage killing protein with similarity to conjugation protein  Mercuric resistance operon regulatory protein  Mercuric resistance protein MerC  Multidrug transporter activation protein  N-acetylmannosamine kinase  N-acetylneuraminate epimerase precursor  Periplasmic mercury ion-binding protein  phage baseplate assembly protein V  Phage head completion protein (GPL)  phage lysis regulatory protein, LysB family  Phage lysozyme  Phage protein GP46  Phage regulatory protein CII (CP76)  Phage Tail Protein X  Phage tail tube protein  phage virion morphogenesis protein  Phage-related protein  Phenolic acid decarboxylase subunit D  Plasmid stabilisation system protein  Plasmid stability protein  Polyketide cyclase / dehydrase and lipid transport  Protein involved in initiation of plasmid replication  putative ABC transporter ATP-binding protein  Putative endoribonuclease SymE  Putative general secretion pathway protein I  putative mercuric transport protein  putative mercury resistance protein  Putative NADH-flavin reductase  putative phage head-tail adaptor  putative phage protein (possible DNA packaging)  putative phage terminase, small subunit, P27 family  Relaxosome protein  Ribbon-helix-helix protein, copG family  Ribokinase  sn-glycerol-3-phosphate transport system permease protein UgpA  transposase  Transposase, TnpA family  transposase/IS protein  transposase/IS protein  Type I secretion system membrane fusion protein PrsE  type VI secretion-associated protein, family  Type-1 restriction enzyme R protein  Tyrosine recombinase XerC | 1,5-anhydro-D-fructose reductase  2,3-dihydroxybenzoate-AMP ligase  ACT domain-containing protein  Alpha-hemolysin translocation ATP-binding protein HlyB  ankyrin repeat protein  ATP-dependent Clp protease proteolytic subunit  Bacteriophage CI repressor helix-turn-helix domain protein  Bacteriophage CII protein  Bacteriophage tail assembly protein  Bacteriophage tail assembly protein  Bacteriophage terminase large (ATPase) subunit  Cation efflux system protein CusF precursor  Chaperone protein EcpD precursor  Colicin E1 (microcin) immunity protein  Colicin V secretion protein CvaA  conjugal transfer transcriptional regulator TraJ  Conjugative accessory protein MbeC  Copper resistance protein A precursor  Copper resistance protein B precursor  Copper resistance protein D  Cro  DicB protein  D-inositol 3-phosphate glycosyltransferase  Ferri-bacillibactin esterase BesA  Ferric enterobactin esterase  glycosyltransferase, MGT family  Glycyl-glycine endopeptidase LytM precursor  head assembly protein  Helix-turn-helix  HTH-type transcriptional activator AllS  HTH-type transcriptional regulator GadW  Inner membrane protein YeeR  invasion lipoprotein InvH  IS1 transposase  Isochorismate synthase/isochorismate-pyruvate lyase MbtI  LexA repressor  Linear gramicidin synthase subunit D  Maltose transport system permease protein MalF  muropeptide transporter  N,N'-diacetylchitobiose-specific phosphotransferase enzyme IIA component  Neu5Ac permease  Neu5Ac-binding protein  P22 coat protein - gene protein 5  P22 tail accessory factor  ParA-like protein  ParB/RepB/Spo0J family partition protein  Phage envelope protein  Phage minor tail protein U  phage prohead protease, HK97 family  Phage-related protein  Phospho-2-dehydro-3-deoxyheptonate aldolase, Tyr-sensitive  Plasmid stabilisation system protein  Plasmid stability protein  PQ loop repeat  Prophage CP4-57 integrase  protein YibB  putative ATP synthase SpaL  putative copper-binding protein PcoE precursor  putative DNA-binding protein with PD1-like DNA-binding motif  putative DNA-binding transcriptional regulator  putative HTH-type transcriptional regulator  Putative multidrug export ATP-binding/permease protein  Putative multidrug export ATP-binding/permease protein  putative oxidoreductase  Putative prophage CPS-53 integrase  putative restriction endonuclease  putative type I restriction enzymeP M protein  putative zinc-type alcohol dehydrogenase-like protein YjmD  Regulatory protein PchR  RepFIB replication protein A  replication protein  Replicative DNA helicase  Ribokinase  Ribose import ATP-binding protein RbsA  RNA one modulator  Sel1 repeat  Sensor kinase CusS  Site-specific recombinase XerC  Surfactin synthase thioesterase subunit  Terminase small subunit  thiazolinyl imide reductase  Transposase  Transposase  Type I restriction enzyme EcoR124II R protein  type III restriction-modification system StyLTI enzyme res  type III secretion system protein SpaQ  Type-1A pilin  XapX domain protein |

**Additional Table 8 - Description of the patients and microbiota samples.**

| **Sample**  **ID** | **Diagnosis** | **Patient ID** | **Sex** | **Age, years** | **Localisation** | **Clinical activity** | **Endo-**  **scopic activity** | **Sample type** | **Platform** | **SRA ID** |
| --- | --- | --- | --- | --- | --- | --- | --- | --- | --- | --- |
| KI01 | CD | MPG001 | F | 40 | ileocolitis-perianal | high | 0 | ileal content | SOLiD | SRS1096086 |
| KI01_t | CD | MPG001 | F | 40 | ileocolitis-perianal | high | 0 | ileal content | Ion Torrent | SRS1096464 |
| KI02 | CD | MPG002 | M | 32 | ileocolitis | remission | 3 | ileal content | SOLiD | SRS1096527 |
| KI05_t | CD | MPG005 | M | 47 | ileocolitis | low | 15 | ileal content | Ion Torrent | SRS1097241 |
| KI08 | CD | MPG008 | M | 48 | colitis | low | 4 | ileal content | SOLiD | SRS1097242 |
| KI08_t | CD | MPG008 | M | 48 | colitis | low | 4 | ileal content | Ion Torrent | SRS1097244 |
| KS10 | CD | MPG010 | F | 32 | colitis | low | 4 | stool | SOLiD | SRS1097246 |
| KS10_t | CD | MPG010 | F | 32 | colitis | low | 4 | stool | Ion Torrent | SRS1097263 |
| KI10 | CD | MPG010 | F | 32 | colitis | low | 4 | ileal content | SOLiD | SRS1097721 |
| KI10_t | CD | MPG010 | F | 32 | colitis | low | 4 | ileal content | Ion Torrent | SRS1097712 |
| KI11_t | CD | MPG011 | M | 23 | ileocolitis | low | 13 | ileal content | Ion Torrent | SRS1097741 |
| KS15 | CD | MPG015 | F | 33 | ileitis | low | 10 | stool | SOLiD | SRS1097809 |
| KI15 | CD | MPG015 | F | 33 | ileitis | low | 10 | ileal content | SOLiD | SRS1099444 |
| KS21 | CD | MPG021 | M | 32 | ileitis-jejunitis | high | 9 | stool | SOLiD | SRS1103557 |
| KS23 | CD | MPG023 | F | 37 | ileocolitis | medium | 14 | stool | SOLiD | SRS1109845 |
| KI23 | CD | MPG023 | F | 37 | ileocolitis | medium | 14 | ileal content | SOLiD | SRS1109854 |
| KS24 | CD | MPG024 | M | 22 | ileocolitis | remission | 8 | stool | SOLiD | SRS1109860 |
| KI24 | CD | MPG024 | M | 22 | ileocolitis | remission | 8 | ileal content | SOLiD | SRS1109912 |
| KI26 | CD | MPG026 | M | 9 | ileocolitis | high | 12 | ileal content | SOLiD | SRS1097800 |
| KS27 | CD | MPG027 | F | 9 | ileocolitis | low | 7 | stool | SOLiD | SRS1109913 |
| KI27 | CD | MPG027 | F | 9 | ileocolitis | low | 7 | ileal content | SOLiD | SRS1109914 |
| KI28 | CD | MPG028 | M | 28 | ileitis | medium | 8 | ileal content | SOLiD | SRS1097808 |
| KS33 | CD | MPG033 | F | 18 | ileitis | medium | 9 | stool | SOLiD | SRS1109915 |
| KS99 | CD | XTR099 | M | 9 | ileocolitis | high | 12 | stool | SOLiD | SRS1109924 |
| KI98 | CD | XTR098 | M | 29 | ileocolitis | high | 8 | ileal content | SOLiD | SRS1109933 |
| KI97 | CD | XTR097 | F | 29 | ileocolitis-perianal | low | 6 | ileal content | SOLiD | SRS1109934 |
| KI96 | CD | XTR096 | M | 25 | colitis | low | 5 | ileal content | SOLiD | SRS1109935 |
| KS95 | CD | XTR095 | F | 42 | ileitis | low | 5 | stool | SOLiD | SRS1109938 |

**Additional Table 9 - List of the genomes used for the construction of the *E. coli* pangenome.**

| **Name** | **Link** | **Description** |
| --- | --- | --- |
| Escherichia_coli_042_uid161985 | http://journals.plos.org/plosone/article?id=10.1371/journal.pone.0008801 | Enteroaggregative |
| Escherichia_coli_536_uid58531 | http://jb.asm.org/content/186/10/3086.abstract | UPEC |
| Escherichia_coli_55989_uid59383 | http://www.genome.jp/kegg-bin/show_organism?org=eck | EAEC |
| Escherichia_coli_APEC_O1_uid58623 |  | APEC |
| Escherichia_coli_APEC_O78_uid187277 |  | APEC |
| Escherichia_coli_CFT073_uid57915 | http://www.genome.wisc.edu/sequencing/upec.htm | UPEC |
| Escherichia_coli_E24377A_uid58395 | http://www.genome.jp/kegg-bin/show_organism?org=ecw | ETEC |
| Escherichia_coli_ETEC_H10407_uid161993 |  | ETEC |
| Escherichia_coli_IAI39_uid59381 | http://www.genome.jp/kegg-bin/show_organism?org=ect | ExPEC |
| Escherichia_coli_IHE3034_uid162007 | http://www.genome.jp/kegg-bin/show_organism?org=eih | meningitis |
| Escherichia_coli_JJ1886_uid226103 |  | UPEC |
| Escherichia_coli_LF82_uid161965 |  | Crohn’s disease associated |
| Escherichia_coli_NA114_uid162139 |  | UPEC |
| Escherichia_coli_O103_H2_12009_uid41013 | http://www.ncbi.nlm.nih.gov/pmc/articles/PMC3067316/ | STEC |
| Escherichia_coli_O104_H4_2009EL_2050_uid175905 | http://en.wikipedia.org/wiki/Escherichia_coli_O104:H4 | enteroaggregative |
| Escherichia_coli_O104_H4_2009EL_2071_uid176128 | http://www.ncbi.nlm.nih.gov/pubmed/23133618 | enteroaggregative |
| Escherichia_coli_O104_H4_2011C_3493_uid176127 | http://www.ncbi.nlm.nih.gov/bioproject/81095 | Isolated from US citizen afflicted with HUS after travel to Germany during the 2011 E coli oubreak. |
| Escherichia_coli_O111_H__11128_uid41023 | http://www.genome.jp/dbget-bin/www_bget?gn:T01097 | EHEC |
| Escherichia_coli_O127_H6_E2348_69_uid59343 | http://www.uniprot.org/taxonomy/574521 | EPEC |
| Escherichia_coli_O157_H7_EC4115_uid59091 | http://en.wikipedia.org/wiki/Escherichia_coli_O157:H7 | EHEC |
| Escherichia_coli_O157_H7_EDL933_uid57831 | http://www.genome.jp/dbget-bin/www_bget?gn:ece | EHEC |
| Escherichia_coli_O157_H7_TW14359_uid59235 | http://en.wikipedia.org/wiki/Escherichia_coli_O157:H7 | EHEC |
| Escherichia_coli_O157_H7_uid57781 | http://en.wikipedia.org/wiki/Escherichia_coli_O157:H7 | enterohemorrhagic |
| Escherichia_coli_O26_H11_11368_uid41021 | http://www.genome.jp/kegg-bin/show_organism?org=eoj | EHEC |
| Escherichia_coli_O55_H7_CB9615_uid46655 | http://www.genome.jp/dbget-bin/www_bget?gn:T01190 | EPEC |
| Escherichia_coli_O55_H7_RM12579_uid162153 | http://www.genome.jp/kegg-bin/show_organism?org=elr | EPEC |
| Escherichia_coli_O7_K1_CE10_uid162115 | http://www.genome.jp/kegg-bin/show_organism?org=eoc | meningitis |
| Escherichia_coli_O83_H1_NRG_857C_uid161987 |  | Crohn’s disease associated |
| Escherichia_coli_PMV_1_uid219679 | http://genomea.asm.org/content/1/5/e00913-13.full.pdf | extraintestinal pathogenic |
| Escherichia_coli_S88_uid62979 | http://www.ncbi.nlm.nih.gov/pubmed/19307211 | neonatal meningitis |
| Escherichia_coli_UM146_uid162043 |  | Crohn’s disease associated |
| Escherichia_coli_UMN026_uid62981 | http://www.genome.jp/kegg-bin/show_organism?org=eum | EXPEC |
| Escherichia_coli_UMNK88_uid161991 | http://www.straininfo.net/genomes/42137 | diarrhea |
| Escherichia_coli_UTI89_uid58541 | http://www.genome.jp/dbget-bin/www_bget?gn:T00338 | UPEC |
| Escherichia_coli_Xuzhou21_uid163995 | http://www.genome.jp/kegg-bin/show_organism?org=elx | EHEC |
| H10407 | http://www.genome.jp/dbget-bin/www_bget?gn:T02069 | ETEC |
| HM605 |  | Crohn’s disease associated |
| B354 | http://www.broadinstitute.org/annotation/genome/escherichia_antibiotic_resistance/GenomeDescriptions.html | Avian |
| Escherichia_coli__BL21_Gold_DE3_pLysS_AG__uid59245 |  | LAB - BL21(DE3) is a derivative of E. coli strain B |
| Escherichia_coli__clone_D_i14__uid162049 | http://www.ncbi.nlm.nih.gov/pmc/articles/PMC3203180/#pone.0026907.s002 | dog-associated isolate, UPEC |
| Escherichia_coli__clone_D_i2__uid162047 | http://www.ncbi.nlm.nih.gov/pmc/articles/PMC3203180/#pone.0026907.s002 | No symptoms, dog UTI-related |
| Escherichia_coli_ABU_83972_uid161975 | http://iai.asm.org/content/74/1/615.short | asymptomatic bacteriuria |
| Escherichia_coli_ATCC_8739_uid58783 | http://www.ncbi.nlm.nih.gov/bioproject/18083 | lacking porin ompC |
| Escherichia_coli_B_REL606_uid58803 | http://www.ncbi.nlm.nih.gov/pubmed/19786035 | evolution experiment |
| Escherichia_coli_BL21_DE3__uid161947 |  | LAB - BL21(DE3) is a derivative of E. coli strain B |
| Escherichia_coli_BL21_DE3__uid161949 | http://www.ncbi.nlm.nih.gov/bioproject/20713 | LAB - BL21(DE3) is a derivative of E. coli strain B |
| Escherichia_coli_BW2952_uid59391 | http://www.genome.jp/kegg-bin/show_organism?org=ebw | LAB K12 derivative |
| Escherichia_coli_DH1_uid161951 |  | LAB |
| Escherichia_coli_DH1_uid162051 | http://openwetware.org/wiki/E._coli_genotypes#DH1 | LAB K12 derivative |
| Escherichia_coli_ED1a_uid59379 | http://www.genome.jp/dbget-bin/www_bget?gn:T00827 | commensal |
| Escherichia_coli_HS_uid58393 | http://www.genome.jp/kegg-bin/show_organism?org=ecx | commensal |
| Escherichia_coli_IAI1_uid59377 | http://www.genome.jp/dbget-bin/www_bget?gn:T00828 | commensal strain |
| Escherichia_coli_K_12_substr__DH10B_uid58979 |  | LAB K-12 |
| Escherichia_coli_K_12_substr__MDS42_uid193705 | http://epa.gov/biotech_rule/pubs/fra/fra004.htm | LAB k12 debilitated strain which does not normally colonize the human intestine. |
| Escherichia_coli_K_12_substr__MG1655_uid57779 | http://www.ncbi.nlm.nih.gov/bioproject/225 | LAB - Non-pathogenic strain MG1655 approximates wild-type E. coli as it has been maintained with very little genetic manipulation except for the curing (removal) of bacteriophage lambda and the F plasmid. |
| Escherichia_coli_K_12_substr__W3110_uid161931 |  | LAB k12 |
| Escherichia_coli_KO11FL_uid162099 | http://www.ncbi.nlm.nih.gov/pubmed/22075923 | ethanol producing, lab derivative of W, |
| Escherichia_coli_KO11FL_uid52593 | http://www.ncbi.nlm.nih.gov/pubmed/22075923 | ethanol producing derivative of W |
| Escherichia_coli_LY180_uid219461 | http://www.ncbi.nlm.nih.gov/pubmed/22504824 | ethanologenic |
| Escherichia_coli_P12b_uid162061 | http://www.biomedcentral.com/1471-2180/5/4#B3 | flagellar H4 producing reference strain, same serotype as STEC |
| Escherichia_coli_SE11_uid59425 | http://www.genome.jp/kegg-bin/show_organism?org=ecy | commensal |
| Escherichia_coli_SE15_uid161939 | http://www.genome.jp/kegg-bin/show_organism?org=ese | commensal |
| Escherichia_coli_SMS_3_5_uid58919 | http://www.genome.jp/kegg-bin/show_organism?org=ecm | multidrug-resistant, environmental |
| Escherichia_coli_W_uid162011 |  | LAB - one of four strains designated as safe for laboratory purposes |
| Escherichia_coli_W_uid162101 | http://www.biomedcentral.com/1471-2164/12/9 | safe for lab use, was originally isolated from the soil of a cemetery near Rutgers University |
| KLY | http://www.ncbi.nlm.nih.gov/pubmed/25043002 |  |
| KTE142 | http://www.ncbi.nlm.nih.gov/biosample/SAMN00854674 |  |
| KTE233 | http://www.ebi.ac.uk/ena/data/view/GCA_000351325.1 | UPEC |
| MC4100 |  | LAB K-12 |
| N1 | http://www.ncbi.nlm.nih.gov/protein/EKI48681.1 | Genomic anatomy of Escherichia coli O157:H7 outbreaks |
| Nissle_1917 | http://www.microbecolhealthdis.net/index.php/mehd/article/viewFile/7512/8855 | probiotic |
| RCE01 |  | this study |
| RCE02 |  | this study |
| RCE03 |  | this study |
| RCE04 |  | this study |
| RCE05 |  | this study |
| RCE06 |  | this study |
| RCE07 |  | this study |
| RCE08 |  | this study |
| RCE10 |  | this study |
| RCE11 |  | this study |

**Additional Table 10 - *E. coli* accessory genome data extraction statistics.**

| Number of the genes in the 81 reference genomes | 394,628 |
| --- | --- |
| Number of the orthology groups (OG) | 8,954 |
| Number of the high-similarity hits produced during alignment of all representatives of OGs against gut microbial gene catalog | 9,182 |
| Number of the unique hits | 9,125 |
| Number of the OGs included in the pangenome | 5,598 |
| Number of the OGs included in the accessory genome (AG) | 2,993 |

**Additional Table 11 – List of virulence-associated *E. coli* genes identified in the set of accessory genes.**

| **Gene ID from Joensen et al, 2014** | ***E. coli* accessory gene ID**  **(as in 3.3 mln microbial gene catalogue)** |
| --- | --- |
| astA:1:AF161000 | GL0062165_O2_scaffold38109_1_6658_7782 |
| celb:1:X63621 | GL0082075_MH0002_scaffold10017_4_921_1097 |
| cnf1:1:U42629 | GL0044190_O2_scaffold38043_1_901_3945 |
| gad:1:CU928158 | GL0080744_O2_scaffold28404_1_5_1162 |
| iha:1:CP000970 | GL0060521_O2_scaffold26551_2_489_2579 |
| ireA:1:CU928162 | GL0042501_V1_scaffold39410_1_1360_3408 |
| iss:1:DQ381420 | GL0044083_V1_scaffold19968_1_53_361 |
| lpfA:1:KC207123 | GL0046691_MH0079_scaffold54255_1_141_650 |
| mchB:1:AE014075 | GL0029215_O2_scaffold23942_3_4454_4747 |
| mchC:1:AJ515251 | GL0029214_O2_scaffold23942_3_2632_4182 |
| mchF:1:AF063590 | GL0022011_MH0008_scaffold7037_4_9656_11521 |
| mcmA:1:CP000247 | GL0059010_O2_scaffold23942_2_195_473 |
| pic:1:AE014075 | GL0062164_O2_scaffold38109_1_1540_5655 |
| sat:1:CP003034 | GL0093073_O2_scaffold4546_2_2075_5851 |
| senB:1:CP001062 | GL0014846_V1_scaffold27068_5_2338_3450 |
| vat:1:X16664 | GL0058871_O2_scaffold5421_3_30694_34824 |
| eilA:1:FN554766 | GL0060866_MH0025_C3696794_1_3029_4726 |

## Additional figures


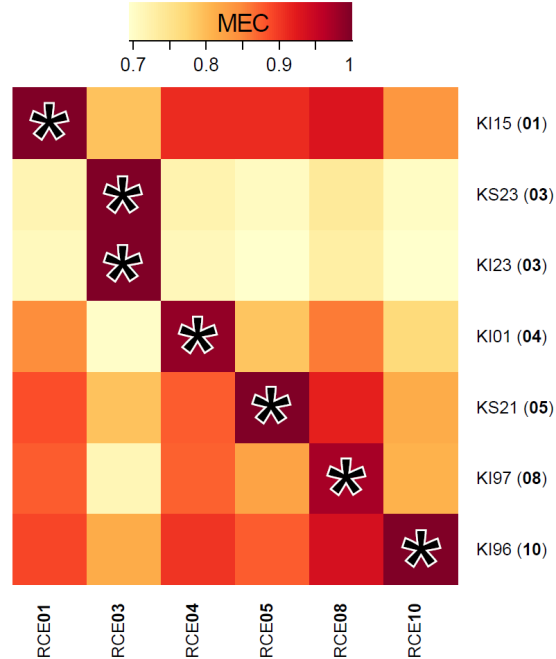


**Additional Figure 1 - Maximum expected coverage (MEC) - fraction of genome length to which the coverage is predicted to converge during mapping of metagenomic reads to genomes - assessed for samples from 6 patients. All 7 metagenomes yielding sufficient coverage of *E. coli* were considered. Columns and rows correspond to genomes and metagenomes, respectively; identical numbers in bold reflect identical patient. Genome most similar to each metagenome is marked with asterisk.**

**
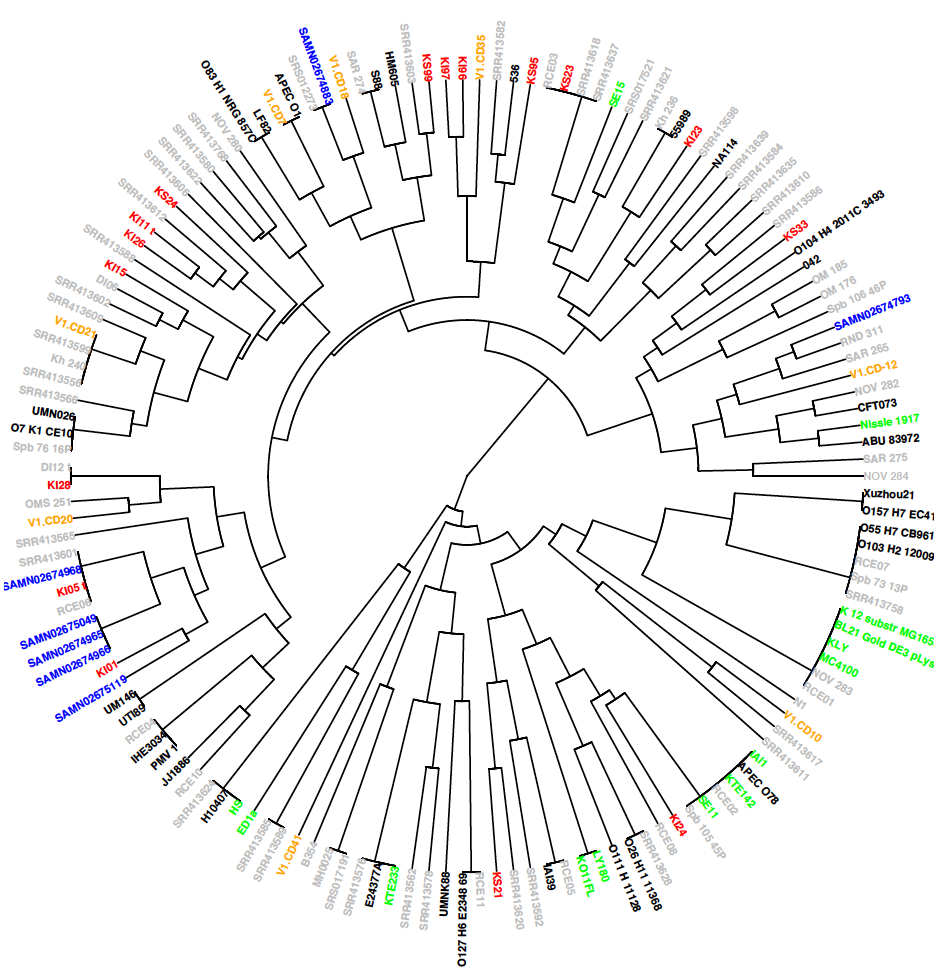
**

**Additional Figure 2 - Clustering of the metagenomes and E. coli genomes based on a unified representation of the 17 virulence-associated genes presence profile. The denotations are similar to ones on Figure 4.**

**A)**

**
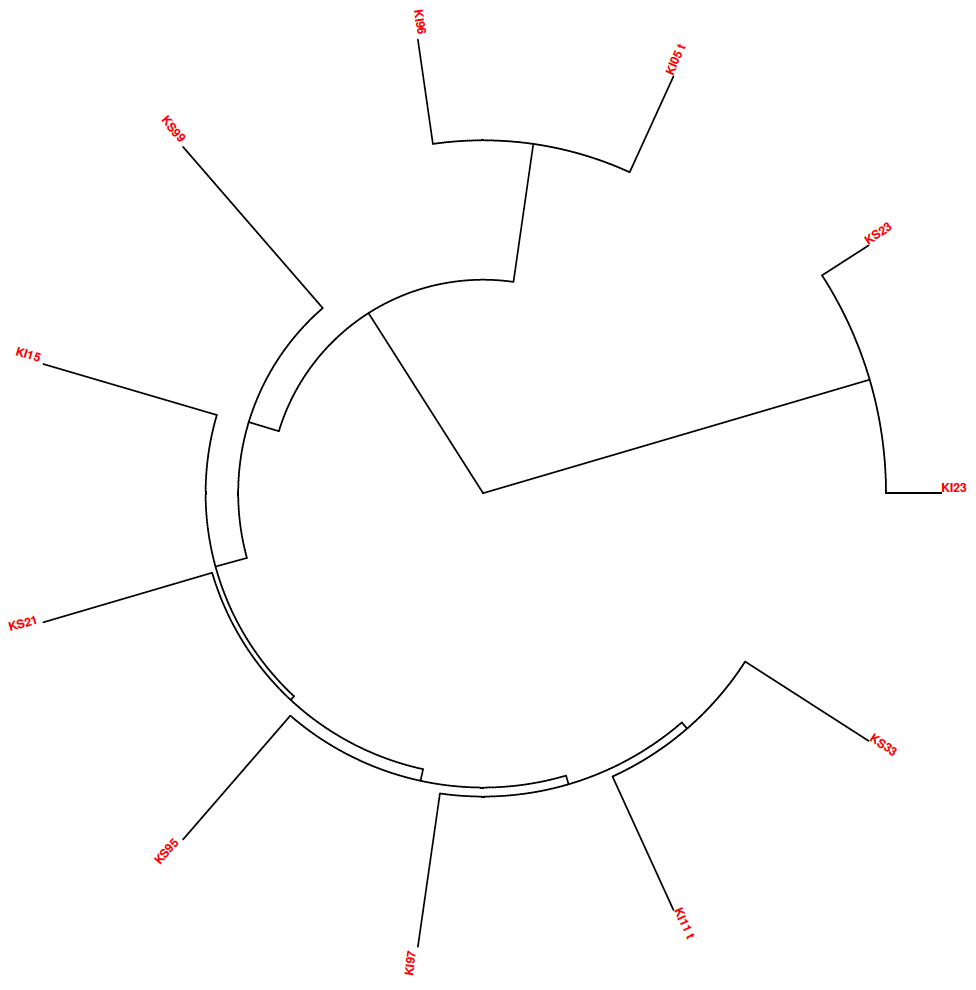
**

**B)**

**
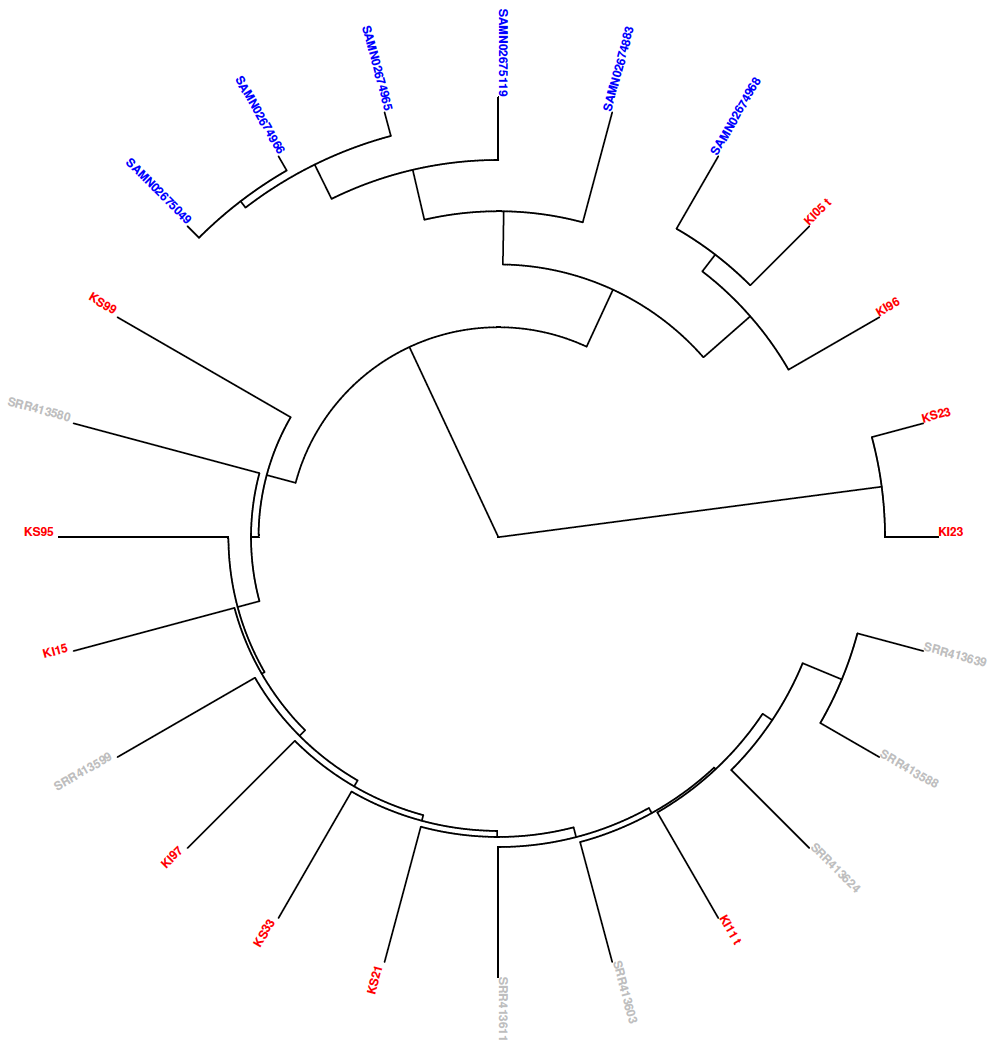
**

**Additional Figure 3 - Cluster analysis of reconstructed E. coli gene content for selected samples yielded using PanPhlAn. A) Samples from Russian CD patients. B) Samples from Russian and selected US CD patients, as well as from selected Chinese healthy controls.**

**
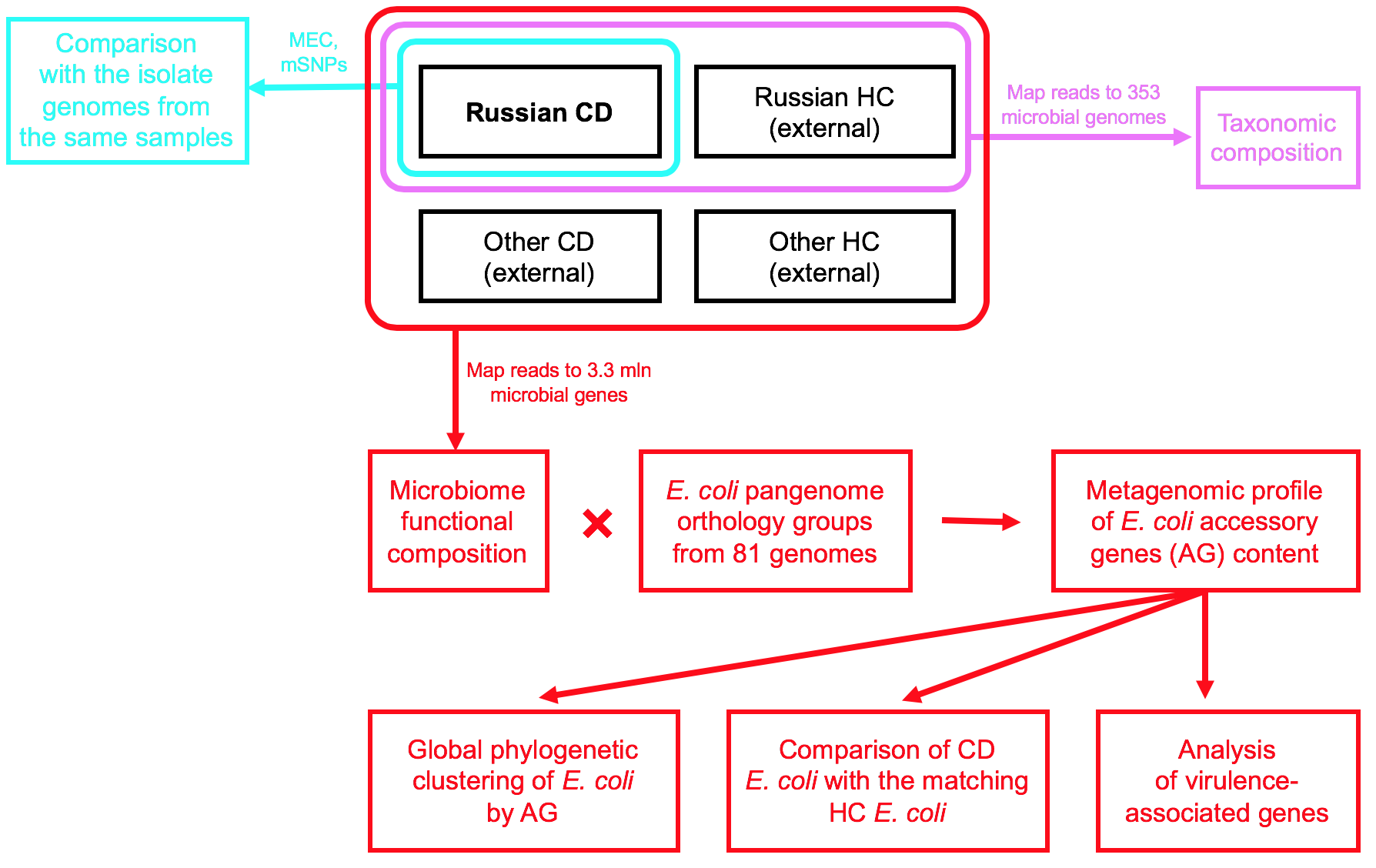
**

**Additional Figure 4 – General workflow of the analysis. Abbreviations: AG – accessory genes, CD – Crohn’s disease, HC – healthy controls, mSNPs – metagenomic SNPs.**
